# Pn-AqpC-Mediated Fermentation Pattern Coordination with the Two-Component System 07 Regulates Host N-Glycan Degradation of Streptococcus pneumoniae

**DOI:** 10.1128/spectrum.02496-22

**Published:** 2022-09-15

**Authors:** Kaiqiang Shen, Qingqing Hu, Lin Zhu, Wenshuang Miu, Yuzhu Dong, Fu Ren, Xiuzhu Dong, Huichun Tong

**Affiliations:** a State Key Laboratory of Microbial Resources, Institute of Microbiology, Chinese Academy of Sciences, Beijing, China; b University of Chinese Academy of Sciences, Beijing, China; c School of Basic Medicine, Shenyang Medical College, Shenyang, China; South China Sea Institute of Oceanology

**Keywords:** *Streptococcus pneumoniae*, oxygen facilitator Pn-AqpC, homolactic acid fermentation, two-component system 07, N-glycan degradation, formate

## Abstract

The opportunistic pathogen Streptococcus pneumoniae (pneumococcus) is a human nasopharyngeal commensal, and host N-glycan metabolism promotes its colonization and invasion. It has been reported that glucose represses, while fetuin, a glycoconjugated model protein, induces, the genes involved in N-glycan degradation through the two-component system TCS07. However, the mechanisms of glucose repression and TCS07 induction remain unknown. Previously, we found that the pneumococcal aquaglyceroporin Pn-AqpC facilitates oxygen uptake, thereby contributing to the antioxidant potential and virulence. In this study, through Tandem Mass Tag (TMT) quantitative proteomics, we found that the deletion of Pn-*aqpC* caused a marked upregulation of 11 proteins involved in N-glycan degradation in glucose-grown pneumococcus R6. Both quantitative RT-PCR and GFP fluorescence reporters revealed that the upregulation of N-glycan genes was completely dependent on response regulator (RR) 07, but not on the histidine kinase HK07 of TCS07 or the phosphoryl-receiving aspartate residue of RR07 in ΔPn-*aqpC*, indicating that RR07 was activated in an HK07-independent manner when Pn-AqpC was absent. The deletion of Pn-*aqpC* also enhanced the expression of pyruvate formate lyase and increased formate production, probably due to reduced cellular oxygen content, indicating that a shunt of glucose catabolism to mixed acid fermentation occurs. Notably, formate induced the N-glycan degradation genes in glucose-grown R6, but the deletion of *rr07* abolished this induction, indicating that formate activates RR07. However, the induction of N-glycan degradation proteins reduced the intraspecies competition of R6 in glucose. Therefore, although N-glycan degradation promotes pneumococcal pathogenesis, the glucose metabolites-based RR07 regulation reported here is of importance for balancing growth fitness and the pathogenicity of pneumococcus.

**IMPORTANCE** Pneumococcus, a human opportunistic pathogen, is capable of metabolizing host complex N-glycans. N-glycan degradation promotes pneumococcus colonization in the nasopharynx as well as invasion into deeper tissues, thus significantly contributing to pathogenesis. It is known that the two-component system 07 induces the N-glycan metabolizing genes; however, how TCS07 is activated remains unknown. This study reveals that formate, the anaerobic fermentation metabolite of pneumococcus, is a novel activator of the response regulator (RR) 07. Although the high expression of N-glycan degradation genes promotes pneumococcal colonization in the nasopharynx and pathogenesis, this reduces pneumococcal growth fitness in glucose as indicated in this work. Notably, the presence of Pn-AqpC, an oxygen-transporting aquaglyceroporin, enables pneumococcus to maintain glucose homolactic acid fermentation, thus reducing formate production, maintaining RR07 inactivation, and controlling N-glycan degrading genes at a non-induced status. Thus, this study highlights a novel fermentation metabolism pattern linking TCS-regulated carbohydrate utilization strategies as a trade-off between the fitness and the pathogenicity of pneumococcus.

## INTRODUCTION

Streptococcus pneumoniae (pneumococcus) is an opportunistic pathogen and is a common cause of community acquired pneumonia, septicemia, and meningitis (1–3). Infections caused by pneumococcus lead to over one million deaths annually (4). Pneumococcus usually asymptomatically colonizes the human nasopharynx and upper respiratory tract, while it can also spread to the lower respiratory tract and other organs, resulting in invasive diseases when the human immune system is compromised (5, 6). To switch successfully from commensal to pathogen, pneumococcus flexibly modulates its metabolic patterns and expression of virulence genes to adapt to changes in the host environment’s pH, oxygen levels, and alternative carbohydrate sources, including N-glycan attached to glycosylated proteins in the nasopharynx, galactose on the host cell surface, and glucose in the blood (7–11). The capability to catabolize these sugars enables pneumococcus to survive in distinct host niches and invade into hosts (12, 13). Therefore, the regulatory mechanisms in carbohydrate metabolism play important roles in the transition of pneumococcus from commensal to pathogenic status (14).

Pneumococcus is a Gram-positive, facultative, anaerobic bacterium that ferments glucose homogenously to lactic acid (i.e., performs homolactic acid fermentation) (15). It obtains energy exclusively from sugar fermentation (16). Consistently, a remarkably large percentage of the pneumococcal genome is comprised of the genes involved in the uptake and metabolism of a variety of sugars, and pneumococcus is capable of using more than 30 different types of sugar (17). In particular, as a pathogen exclusively infecting humans, pneumococcus is capable of utilizing the complex N-glycans attached to the N-glycosylated proteins on human cell surfaces (12). The metabolism of N-glycans not only provides pneumococcus with a carbon source but also exposes the receptors on the host cell surfaces, thus promoting its invasion of host tissues (18). In fact, genes encoding the proteins involved in N-glycan metabolism have been identified in the pneumococcal genome (18–21), and these proteins participate in the degradation and transport of the host N-glycans into the pneumococcal cells as well as in the catabolism of these carbohydrates. Recently, it was found that these genes are induced by fetuin, a glycoconjugated model protein, via a two-component signal transduction system (TCS) 07, but they are repressed by glucose (20). However, how TCS07 induction and glucose suppress the host N-glycan metabolism in S. pneumoniae remains unknown.

In a previous study, we found that the pneumococcal Pn-AqpC protein facilitates O_2_ permeation into cells and promotes H_2_O_2_ production and antioxidant abilities, thereby contributing to the virulence of pneumococcus (22). Unexpectedly, a Tandem Mass Tag (TMT) proteomics analysis revealed that in the presence of glucose, the deletion of Pn-*aqpC* caused remarkable increases in the abundance of the 11 proteins involved in N-glycan degradation. However, this upregulation was completely abrogated when RR07, but not HK07, was absent, implying that RR07 was involved in the induction of N-glycan degradation proteins but was independent of phosphorylation by HK07. The deletion of Pn-*aqpC* also elevated pyruvate formate lyase (PFL) and increased formate production. Supplementation with sodium formate induced the genes involved in N-glycan degradation in the wild-type but not in the Δ*rr07* strain, indicating that formate induction of the N-glycan degradation genes occurred through RR07. Therefore, this work revealed that the metabolite formate could activate RR07 and that pneumococcus controls glucose catabolism homogenously to lactic acid in the presence of Pn-AqpC. So, keeping RR07 inactivated leads to the N-glycan degradation genes not being induced, and this metabolite-mediated carbohydrate utilization regulation confers intraspecies competitive fitness to pneumococcus.

## RESULTS

### TMT quantitative proteomics analysis reveals increased abundances of the proteins for N-glycan degradation and mixed acid fermentation in the absence of Pn-AqpC.

**FIG 1:**
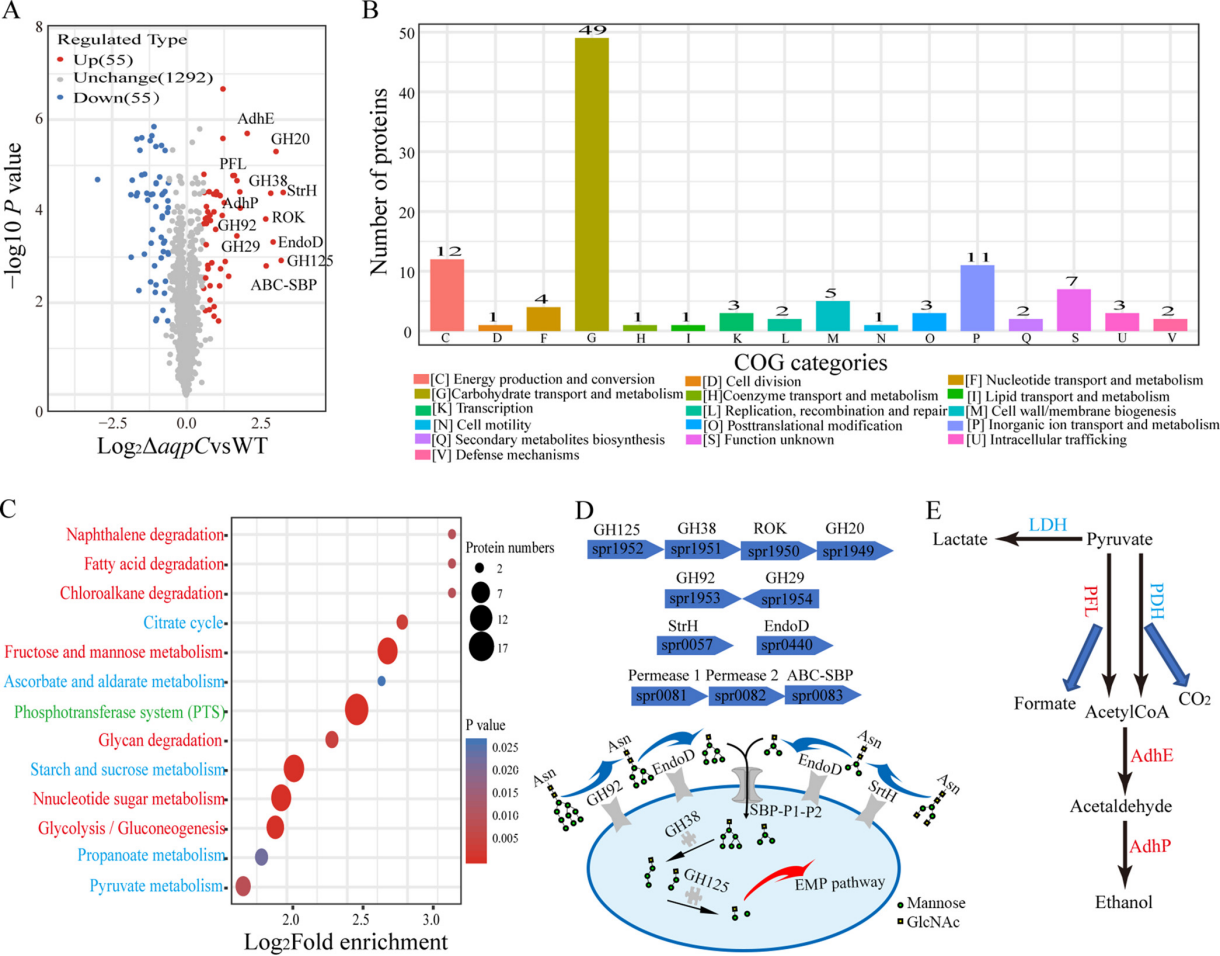
A TMT quantitative proteomics assay revealed that Pn-*aqpC* deletion caused differentially expressed proteins in pneumococcus R6. (A) The volcano plot of differentially expressed proteins in ΔPn-*aqpC*. Beside the dots are the names for the top 20% upregulated proteins. Inside the parentheses are the numbers of upregulated (red dots), downregulated (blue dots), and unchanged (gray dots) proteins in ΔPn-*aqpC*. (B) COG category analysis for differentially expressed proteins in the ΔPn-*aqpC* mutant. Numbers of proteins falling into each category are shown at the tops of the columns. (C) KEGG pathway enrichment analysis of the differentially expressed proteins in the ΔPn-*aqpC* mutant. The KEGG pathway database was used to identify the enriched pathways via a two-tailed Fisher’s exact test to test the enrichment of differentially expressed proteins against all identified proteins. A pathway with a *P* value of <0.05 is considered statistically significant. The dot colors represent the significance of the *P* value, and the dot diameters indicate the differentially expressed protein numbers in each functional pathway. The enriched pathways in red, blue and green characters are participated in by up-, down- and both up- and down-regulated proteins, respectively. (D) Schematics of organization of the R6 genes encoding the host N-glycan degradation proteins that were upregulated in the ΔPn-*aqpC* mutant (upper panel). The bullets show the gene directions, with the gene accession numbers inside and the protein names on the top, respectively. The lower panel shows a working model of N-glycan degrading proteins. (E) Carbohydrate metabolic shunts via pyruvate in the pneumococcal Pn-*aqpC* deletion mutant. The upregulated and downregulated proteins in ΔPn-*aqpC* are shown in red and blue, respectively. LDH, lactate dehydrogenase; PFL, pyruvate formate-lyase; PDH, pyruvate dehydrogenase complex; AdhE, aldehyde-alcohol dehydrogenase; AdhP, alcohol dehydrogenase.

To explore the physiological roles of the pneumococcal Pn-AqpC, a TMT quantitative proteomics analysis was conducted to identify the differentially expressed proteins in strain R6 and in its Pn-*aqpC* deletion mutant. The two strains were grown statically in brain heart infusion (BHI) broth, and cells in the mid-exponential-phase were collected and subjected to a TMT proteomics analysis. In total, 1,402 proteins were identified in these two strains (Data Set S1). Using a >1.5-fold change and a *P* value of <0.05 as a cutoff value, 110 proteins were identified as being differentially expressed due to Pn-*aqpC* deletion, with 55 proteins being upregulated and 55 proteins being downregulated (Fig. 1A; Data Set S1). A Clusters of Orthologous Groups (COG) of proteins analysis indicated that the differentially expressed proteins fell into 16 functional categories, with 49 proteins (45%) being involved in carbohydrate transport and metabolism and 12 proteins (11%) being involved in energy production and conversion (Fig. 1B), indicating that Pn-AqpC plays important roles in modulating energy metabolism based on its oxygen transporter function. A Kyoto Encyclopedia of Genes and Genomes (KEGG) pathway enrichment analysis demonstrated that among these upregulated proteins, those participating in host glycan degradation, glycolysis/gluconeogenesis, and fructose/mannose metabolism were overrepresented, while among the downregulated proteins, those involved in pyruvate metabolism, the phosphotransferase system (PTS), and the pentose phosphate pathway were overrepresented (Fig. 1C). Notably, the most upregulated (6 to 9-fold) proteins in the ΔPn-*aqpC* strain were those involved in N-glycan degradation (Fig. 1D; Data Set S1), including alpha-1,6-mannosidase (GH125), alpha-1,3-mannosidase (GH38), ROK family protein (ROK), and hexosaminidase (GH20), which are encoded by a four-gene operon spr1952-spr1951-spr1950-spr1949, as well as alpha-1,2-mannosidase (GH92), alpha-1,3/4-fucosidase (GH29), Beta-N-acetyl-hexosaminidase (StrH), and endo-beta-N-acetylglucosaminidase (EndoD), which are encoded by the genes spr1953, spr1954, spr0057, and spr0440, respectively, as well as a sugar-transporting ABC transporter encoded by the operon spr0081-spr0082-spr0083. In addition, the deletion of Pn-*aqpC* caused the 1.8-fold and 1.6-fold downregulation, respectively, of the lactate dehydrogenase (LDH) and pyruvate dehydrogenase (PDH) complexes, which consist of a TPP-dependent acetoin dehydrogenase beta chain (AcoB) and an alpha chain (AcoA), respectively, and also caused the 3.1-fold, 4.1-fold, and 3.5-fold upregulation of pyruvate formate-lyase (PFL), aldehyde-alcohol dehydrogenase (AdhE), and alcohol dehydrogenase (AdhP), respectively (Fig. 1E; Data Set S1). This suggested that the deletion of Pn-*aqpC* might alter the pneumococcal carbohydrate metabolic flux from homolactic to mixed acid fermentation.

### The deletion of Pn*-aqpC* increases the transcription and translation of N-glycan degradation genes.

**FIG 2:**
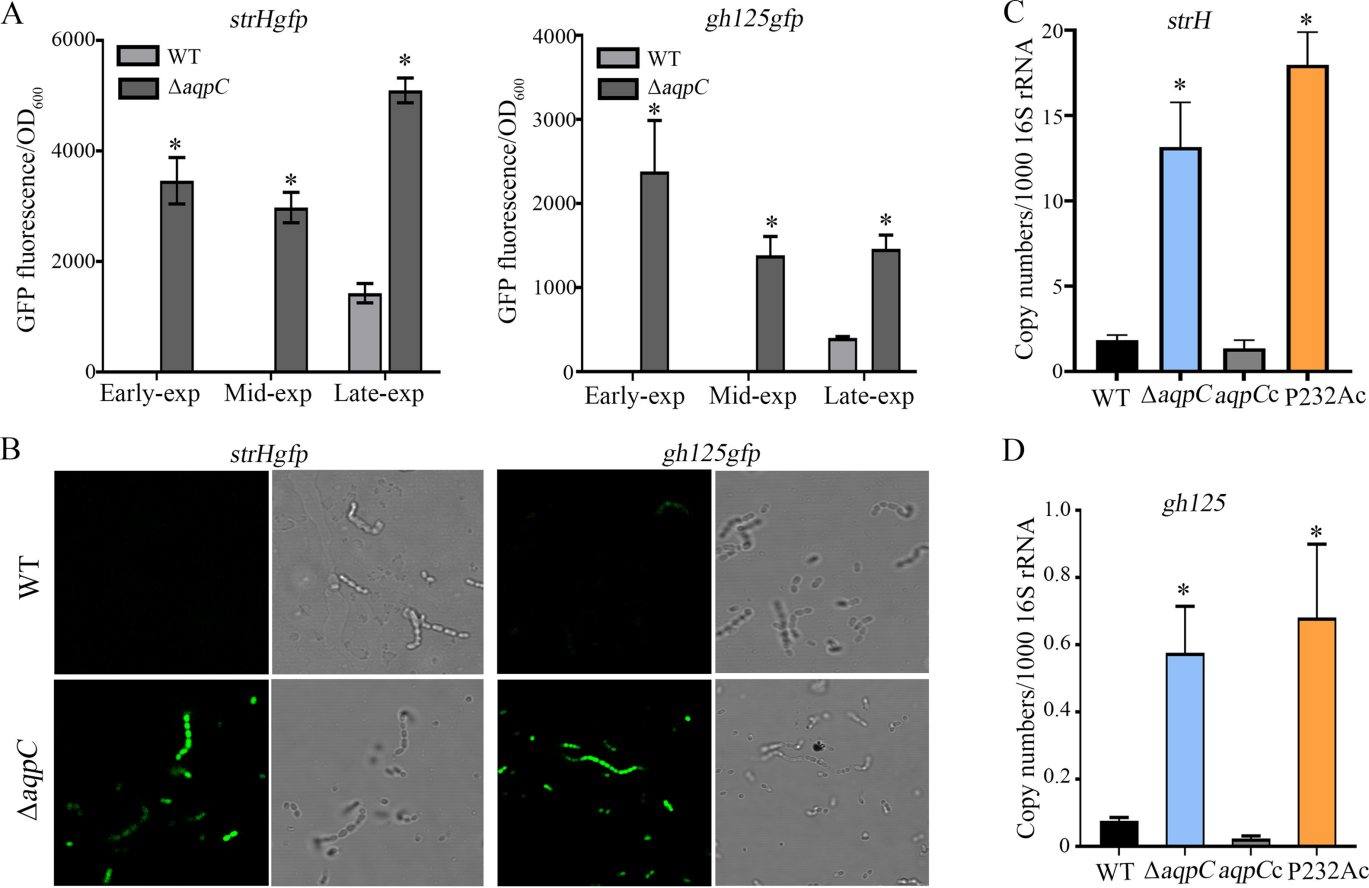
Deletion of Pn-*aqpC* increases translation (A and B) and transcription (C and D) of the *strH* and *gh125* genes encoding N-glycan degradation proteins in pneumococcus R6. (A) The superfolding green fluorescent protein gene sf-*gfp* was fused with the C terminus of *strH* and *gh125* in wild-type (WT) and Pn-*aqpC* deletion (Δ*aqpC*) strains, respectively, to construct *strHgfp* (left panel) and *gh125gfp* (right panel) reporter strains. The four strains were grown in BHI broth, and the optical density at 600 nm (OD_600_) and GFP fluorescence were measured for early-, mid-, and late-exponential cultures. The results are expressed as GFP fluorescence/OD_600_. (B) The strains in panel A were grown in BHI broth, and cells in the late-exponential phase were collected and washed twice with PBS. After 30 min of air exposure in the dark, cells were examined using confocal laser microscopy at an excitation wavelength of 488 nm and an emission wavelength from 500 to 600 nm (left). The corresponding bright field contrast images are shown at the right. (C and D). The wild-type (WT), ΔPn-*aqpC* (Δ*aqpC*), Pn-*aqpC* complementary (*aqpC*c), and P232A mutated Pn-*aqpC* complementary (P232Ac) strains were grown in BHI broth, and mid-exponential cells were collected for total RNA extraction. qRT-PCR was implemented to quantify the transcript copies of *strH* (C) and *gh125* (D). The results are expressed as transcript copy numbers per 1,000 16S rRNA. For panels A, C, and D, the experiments were repeated three times with triplicate samples for each experiment. The averages ± standard deviations from three independent experiments are shown. * Significantly different from the wild-type strain, one way analysis of variance (ANOVA); Tukey’s *post hoc* test (*P* < 0.05). For panel B, the experiments were repeated three times, and representative pictures are shown.

To verify the upregulation of the N-glycan degradation proteins in the Pn-*aqpC* deletion mutant, we selected beta-N-acetylhexosaminidase (StrH) and alpha-1,6-mannosidase (GH125) as objects, based on their highest upregulation relative to others in the ΔPn-*aqpC* strain, as determined by the TMT proteomics analysis (Data Set S1). In addition, the gene *gh125* was situated in a four-gene operon; thus, the expression levels of GH125 could represent the remaining three genes (Fig. 1D). The GFP reporter strains for StrH and GH125 were constructed by fusing the sf-*gfp* gene, which encodes superfolding green fluorescent protein, to the C terminus of *strH* (spr0057) and *gh125* (spr1952) in the wild-type and Pn-*aqpC* deletion strains, respectively. The two reporter strains were then grown in BHI broth, and the expressions of StrH and GH125 in the early-, mid-, and late-exponential phases were examined. Significantly higher GFP fluorescence intensities for StrH and GH125 were observed in the ΔPn-*aqpC* mutant than in the wild-type strain in all three growth phases (Fig. 2A). In particular, almost no GFP fluorescence could be detected in early-exponential or mid-exponential cells of the wild-type strain. Consistently, confocal laser microscopy detected high GFP fluorescence in mid-exponential ΔPn-*aqpC* cells, while GFP fluorescence was almost invisible in wild-type cells (Fig. 2B). These demonstrated that the absence of Pn-AqpC remarkably induces the expression of these two N-glycan degradation proteins.

To examine whether the deletion of Pn-*aqpC* induced the transcription of *strH* and *gh125*, Quantitative reverse transcription-PCR (qRT-PCR) was implemented. The wild-type, ΔPn-*aqpC* mutant, and Pn-*aqpC* complementary (*aqpC*com) strains were grown in BHI broth, and mid-exponential cells were collected for a qRT-PCR assay. We found 7.2-fold and 7.6-fold higher *strH* and *gh125* transcript abundances in the ΔPn-*aqpC* mutant than in the wild-type strain, respectively, while the Pn-*aqpC* complementary strains recovered the expression of these two genes (Fig. 2C and D). This indicated that in the presence of Pn-AqpC, the transcription of *strH* and *gh125* was repressed by unknown mechanisms. In addition, the deletion of Pn-*aqpC* also induced the transcription of *gh92* (spr1953), *gh29* (spr1954), and spr0081 (Fig. S1), which encode other N-glycan degrading proteins. Thus, consistent with the elevated protein abundances (Fig. 1D), the deletion of Pn-*aqpC* also induced the transcription of the encoding genes. It is worth noting that unlike the complementing wild-type Pn-AqpC protein, a complement with Pn-AqpC carrying an alanine substitution for Pro232 in ΔPn-*aqpC* failed to recover the lower expressions of N-glycan degradation genes compared to the wild-type strain (Fig. 2C and D; Fig. S1). Pro232 was found to be the key amino acid residue in Pn-AqpC for oxygen transportation (22). Thus, this finding indicates that the Pn-AqpC-related induction of N-glycan degradation genes is most likely associated with intracellular oxygen levels.

### The deletion of Pn-*aqpC* induces PFL expression and elevates the mixed acid fermentation of glucose.

**FIG 3:**
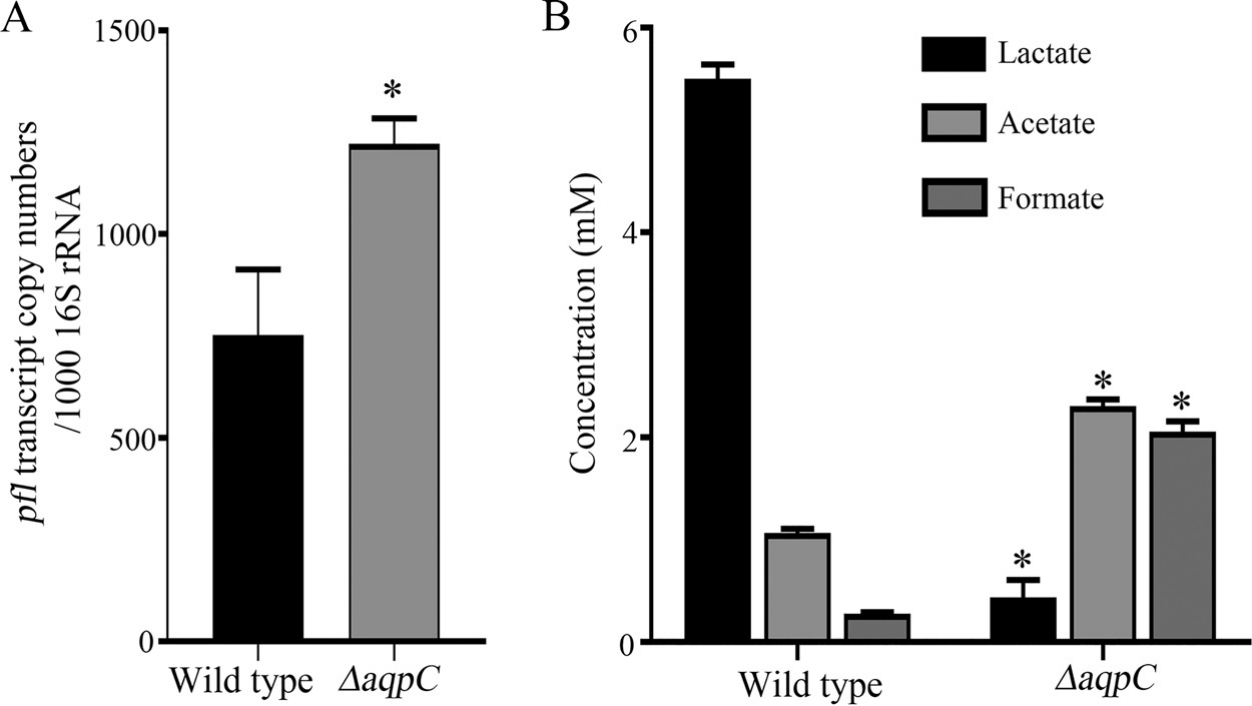
Deletion of Pn-*aqpC* induces *pfl* expression and elevates mix-acid fermentation of glucose of pneumococcus R6. (A) The wild-type and ΔPn-*aqpC* (Δ*aqpC*) strains were grown in BHI broth, and mid-exponential-phase cells were used for total RNA extraction. qRT-PCR was implemented for quantification of the *pfl* transcript. The results are expressed as transcript copy numbers per 1,000 16S rRNA. (B) Early stationary cultures were collected, and lactic, acetic, and formic acids in the spent cultures were measured using high pressure liquid chromatography (HPLC). The experiments were repeated three times with triplicate samples for each experiment. The averages ± standard deviations from three independent experiments are shown. * Significantly different from the datum in the wild-type strain; Student's *t* test (*P* < 0.05).

As we observed a 3.1-fold higher pyruvate formate lyase (PFL) protein abundance in ΔPn-*aqpC* relative to the wild-type strain as determined by the TMT proteomics analysis (Fig. 1E), qRT-PCR was implemented, and a 2-fold higher *pfl* transcription was revealed in the Pn-*aqpC* deletion mutant relative to the wild-type strain (Fig. 3A).

In anaerobic growth, PFL catalyzes the conversion of pyruvate to formate and acetyl-CoA, and the latter is then converted to acetate by phosphate acetyltransferase and acetate kinase (23). Based on the O_2_ transport function of Pn-AqpC (22), we assumed that the elevated PFL abundance in the cellular O_2_-absent Pn-*aqpC* deletion mutant would alter glucose fermentation patterns. Using high performance liquid chromatography (HPLC), we observed 2.1-fold and 7.4-fold higher acetate and formate but 8.7-fold lower lactate in the ΔPn-*aqpC* mutant, relative to the wild-type strain (Fig. 3B). This indicated that in the absence of Pn-AqpC, the glucose metabolic flux in pneumococcus would be shifted from homolactic to mixed acid fermentation by producing more formate and acetate.

### Two-component system response regulator (RR) 07 is involved in the regulation of the N-glycan degradative genes in the absence of Pn-AqpC.

**FIG 4:**
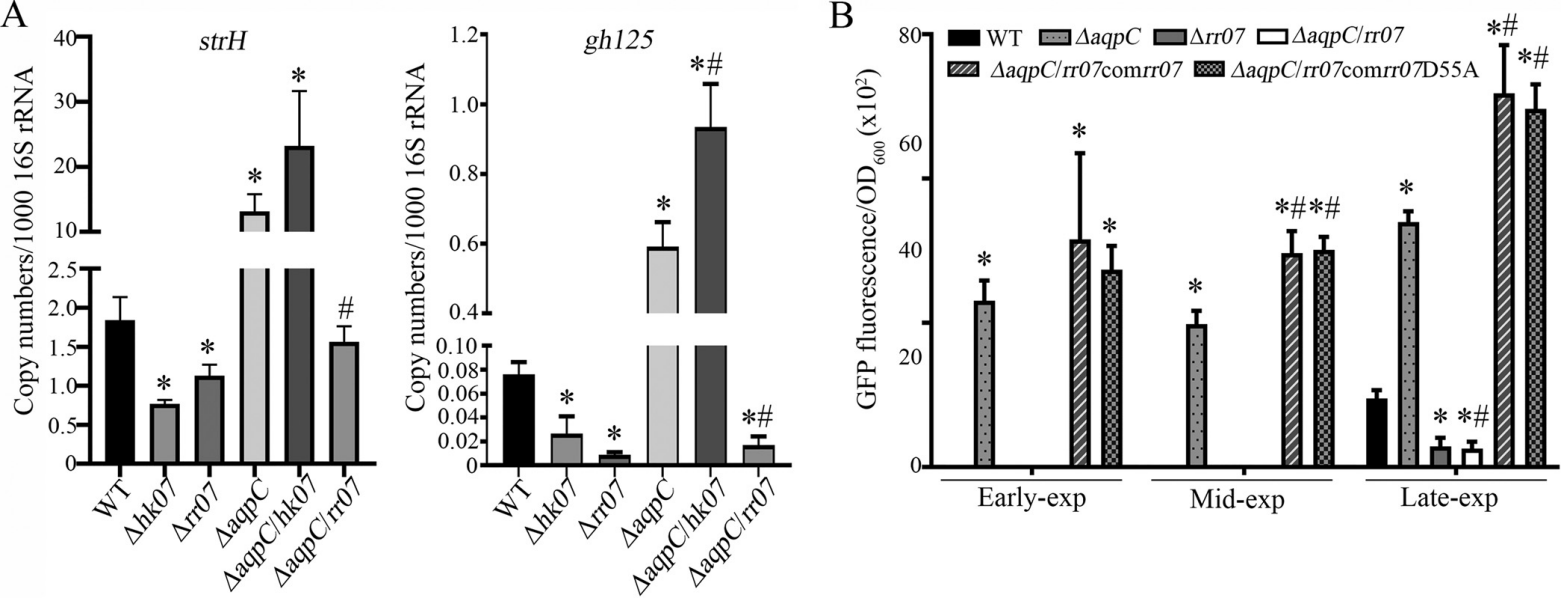
Assays of RR07 involvment in N-glycan degradation gene induction by Pn-*aqpC* deletion. (A) The *rr07* and *hk07* genes were, respectively, deleted in the wild-type and ΔPn-*aqpC* strains to obtained the Δ*hk07*, Δ*rr07*, and ΔPn-*aqpC*/*hk07* (Δ*aqpC*/*hk07*), and the ΔPn-*aqpC*/*rr07* (Δ*aqpC*/*rr07*) strains. These strains, together with the wild-type (WT) and ΔPn-*aqpC* (Δ*aqpC*) strain, were grown in BHI broth, and mid-exponential cells were used for total RNA extraction. Then, qRT-PCR was implemented to determine the transcript copy numbers of the *strH* and *gh125* genes. The results are expressed as transcript copy numbers per 1,000 16S rRNA. (B) The *rr07* gene was deleted in the WT-*strHgfp* (WT) and ΔPn-*aqpC-strHgfp* (Δ*aqpC*) strains to construct strains Δ*rr07*-*strHgfp* (Δ*rr07*), and ΔPn-*aqpC*/*rr07*-*strHgfp* (Δ*aqpC*/*rr07*). In addition, RR07 and RR07D55A were ectopically expressed in strain ΔPn-*aqpC*/*rr07*-*strHgfp* to construct ΔPn-*aqpC*/*rr07*-*strHgfp*com*rr07* (Δ*aqpC*/*rr07*com*rr07*) and ΔPn-*aqpC*/*rr07*-*strHgfp*com*rr07*D55A (Δ*aqpC*/*rr07*com*rr07*D55A). The constructed strains were grown in BHI broth, and aliquots of early-, mid-, and late-exponential cells were measured for optical density at 600 nm (OD_600_) and GFP fluorescence, respectively. The results are expressed as GFP fluorescence/OD_600_. The experiments were repeated three times with triplicate samples for each experiment. The averages ± standard deviations from three independent experiments are shown. For panels A and B, * and # denote significant differences from the data of the wild-type strain and the ΔPn-*aqpC* mutant, respectively, one way ANOVA; Tukey’s *post hoc* test (*P* < 0.05).

Based on a recent report that the two-component system TCS07 activates transcription of the N-glycan degradation genes (20), we further examined whether TCS07 was also involved in the induction of these genes due to Pn-*aqpC* deletion. For this purpose, the *rr07* gene was deleted in the wild-type R6 and ΔPn-*aqpC* strains to construct the Δ*rr07* and ΔPn-*aqpC*/*rr07* double deletion strains. *hk07* was also deleted in these two strains to construct the Δ*hk07* and ΔPn-*aqpC*/*hk07* double deletion strains. Next, the transcript abundances of *strH* and *gh125*, the representative genes for N-glycan degradation, were quantified vie qRT-PCR. As shown in Fig. 4A, the deletion of either *hk07* or *rr07* caused a 1.6 to 8.5-fold decrease in the transcription of *strH* and *gh125* in the wild-type strain, thereby confirming the positive regulation of TCS07 on the N-glycan degradation genes. However, in comparison with the 7.2-fold and 7.6-fold elevated transcriptions of *strH* and *gh125*, respectively, that are caused by Pn-*aqpC* deletion, simultaneously deleting Pn-*aqpC* and *rr07* abolished the induction of *strH* and resulted in an even higher repression of *gh125*. This indicated that the effect of Pn-AqpC on the transcription of the N-glycan degradation genes is via TCS07 regulation, most likely through RR07, as the induced transcriptions of *strH* and *gh125* by the deletion of Pn-*aqpC* were retained when *hk07* was deleted (Fig. 4A).

Next, we examined the effect of RR07 on StrH protein expression. *rr07* was deleted in both the WT-*strHgfp* and ΔPn-*aqpC*-*strHgfp* strains to construct the Δ*rr07*-*strHgfp* and ΔPn-*aqpC*/*rr07*-*strHgfp* strains, respectively. The two strains were grown in BHI broth, and GFP fluorescence was monitored during the whole growth period. Consistent with the decreased transcription of *strH* caused by the deletion of *rr07* from both the wild-type and ΔPn-*aqpC* strains (Fig. 4A), GFP fluorescence was dramatically reduced in early-, mid-, and late-exponential ΔPn-*aqpC*/*rr07*-*strHgfp* cells compared with the ΔPn-*aqpC* cells, and a 3.3-fold decrease in late-exponential Δ*rr07*-*strHgfp* cells was observed, compared with the WT-*strHgfp* cells. Of note, almost the same GFP fluorescence intensity was determined in the ΔPn-*aqpC*/*rr07*-*strHgfp* and the Δ*rr07*-*strHgfp* cells (Fig. 4B). Moreover, ectopically expressed *rr07* via the shuttle plasmid pIB166 recovered GFP fluorescence in the ΔPn-*aqpC*/*rr07*-*strHgfp* strain to even higher levels than that observed in the ΔPn-*aqpC* strain (Fig. 4B). This further verified that the Pn-AqpC-mediated altered expression of the N-glycan degradation genes is in fact regulated by RR07. In contrast, the deletion of Pn-*aqpC* did not alter the transcription of the *rr07* gene (Fig. S2A), indicating that the regulatory effect of RR07 is not due to its expression levels, but rather could be due to protein activation in the absence of Pn-AqpC.

### Activation of RR07 is independent of phosphorylation in the absence of Pn-AqpC.

The classical activation mode of the response regulator in a two-component regulatory system is through a phosphorylation relay, which depends on the phosphorylation at a specific aspartic acid (Asp) residue by its cognate histidine kinase (24). Therefore, we first examined the role of HK07 in the activation of RR07 in the ΔPn-*aqpC* strain. The *hk07* gene was deleted in wild-type and ΔPn-*aqpC* strains to construct Δ*hk07* and ΔPn-*aqpC*/*hk07* strains, respectively, and then the transcripts of *strH* and *gh125* in these two strains were quantified using qRT-PCR. As shown in Fig. 4A, the deletion of *hk07* caused 2.4-fold and 2.8-fold reductions of the *strH* and *gh125* transcripts, respectively, in the wild-type strain, whereas the deletion of *hk07* in the ΔPn-*aqpC* mutant did not affect *strH* transcription and even induced *gh125*, unlike the deletion of *rr07* in the ΔPn-*aqpC* mutant, which recovered *strH* and *gh125* transcriptions to lower levels than in the wild type, respectively. This suggests that the cognate phosphor donor HK07 is dispensable for the activation of RR07 as well as for the increased expression of the N-glycan degradation genes when Pn-AqpC is absent. Consistently, D55A mutated RR07, in which the 55^th^ Asp, a residue accepting the phosphoryl group from the cognate HK07 (25), was mutated into alanine, was still able to complement the expression of *strH* in ΔPn-*aqpC*/*rr07* to even higher levels than in ΔPn-*aqpC* (Fig. 4B).

Next, we examined the phosphorylation status of RR07 in both the wild-type strain and the ΔPn-*aqpC* mutant. A *rr07*-6×His strain was constructed by fusing the 6 Histidine encoding sequence to the C terminus of *rr07* at the genomic loci of the wild-type and ΔPn-*aqpC* strains. Mid-exponential WT-*rr07*-6×His and ΔPn-*aqpC*-*rr07*-6×His cells were then collected, and cellular RR07-6×His proteins were immunoprecipitated (IP) using Anti-His tag MAb-Magnetic Beads (Fig. S3A). These IP pulldown RR07-6×His proteins were first subjected to a Pro-Q Diamond Phosphoprotein Gel Stain (Thermo Fisher Scientific), but no phosphorylated RR07-6×His proteins were detected (Fig. S3B). Next, they were examined using Phos-tag Acrylamide gels, and a known phosphorylated protein casein and the recombinant RR07-6×His protein were included as positive and negative references, respectively (Fig. S3C). However, The Phos-tag Acrylamide gels also failed to reveal a phosphorylated RR07 protein from either the wild-type strain or the ΔPn-*aqpC* mutant strain (Fig. S3C). Consistently, a liquid chromatography-tandem mass spectrometry (LC-MS/MS) analysis did not identify phosphorylation modifications at Ser or Thr residues, which are the potential sites to be phosphorylated by the serine/threonine kinase and regulate the activities of the response regulators (26, 27), of the RR07-6×His proteins IP-pulled down from either the wild-type or ΔPn-*aqpC* strains (Data Set S1). Therefore, the pneumococcal RR07 activation should be independent of phosphorylation and should occur through other (unknown) mechanisms when Pn-AqpC is absent.

### Formate induces N-glycan degradation genes via the activation of RR07.

**FIG 5:**
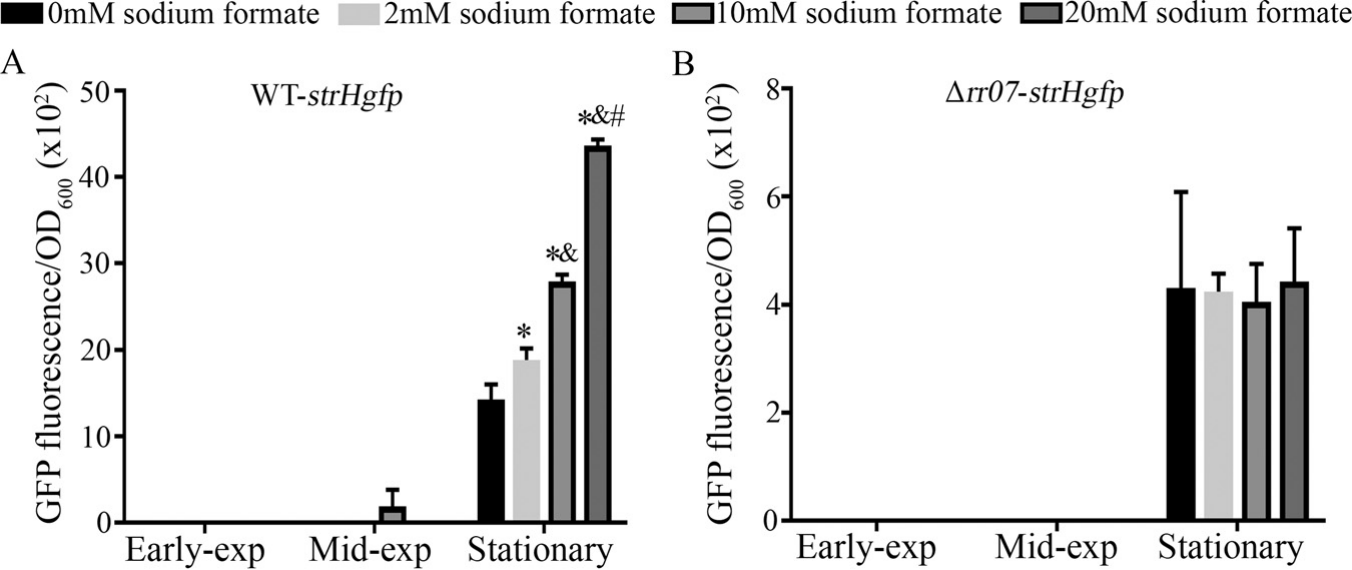
Determination of formate induction on the N-glycan degradation gene *strH* in pneumococcus R6 and its *rr07* deletion strain. The WT-*strHgfp* (A) and Δ*rr07*-*strHgfp* (B) strains were grown in BHI broth with or without supplementation of gradient concentrations of sodium formate. The optical density at 600 nm (OD_600_) and GFP fluorescence of early-exponential, mid-exponential, and stationary cells were measured. The results are expressed as GFP fluorescence/OD_600_. The experiments were repeated three times with triplicate samples included for each experiment. The averages ± standard deviations from three independent experiments are shown. *, &, and # denote significant differences from the data in the wild-type strain without and with 2 mM or 10 mM sodium formate supplementation, respectively, one way ANOVA; Tukey’s *post hoc* test (*P* < 0.05).

Considering that more formate and acetate were produced from glucose fermentation in the absence of Pn-AqpC relative to the wild-type strain (Fig. 3B) and that formate activates the transcriptional regulators CcpA and GlnR (28), we then explored the role of formate in activating RR07 and upregulating the expression of genes encoding the N-glycan degradation proteins. A gradient of sodium formate was supplemented to the BHI-grown WT-*strHgfp* cultures, and the GFP fluorescence intensities of these cultures were measured during growth. As shown in Fig. 5A, compared to WT-*strHgfp* cells grown in BHI broth, the addition of 2 mM, 10 mM, and 20 mM sodium formate increased the GFP fluorescence intensities by 1.5-, 2.1- and 3.0-fold in stationary cells, respectively, indicating that sodium formate induced *strH* expression in the pneumococcus strain R6. The addition of gradient concentrations of sodium acetate or sodium lactate did not change the expression of *strH* (Fig. S4A and B). Of note, formate could only induce N-glycan degradation genes in cells at the late-exponential-phase, not in the earlier growth phases, implying that formate might collaborate with other growth-phase related factors to exert inductive effects.

To further examine whether the formate induction of *strH* was related to RR07, the same gradient of concentrations of sodium formate was added to the Δ*rr07*-*strHgfp* cultures. GFP fluorescence measurement during the growth (Fig. 5B) indicated that sodium formate did not induce *strH* expression when *rr07* was deleted, indicating that the formate induction of *strH* should occur via RR07. Since the addition of formate did not alter the transcription of *rr07* in WT-*strHgfp* (Fig. S2B), this indicated that formate acts as an activator of RR07 and thereby induces N-glycan degrading genes.

### Low expression of the N-glycan degradation genes confers growth fitness to pneumococcus in glucose.

Given that the N-glycan degradation genes were repressed in the glucose grown wild-type strain R6 (Fig. 2), to explore the physiological significance of the repression of the N-glycan degradation genes, we first constructed a strain highly expressing these genes by overexpressing RR07, the positive regulator of N-glycan degradation genes, as indicated in Fig. 4A. An approximately 17-fold increased expression of *strH* was observed in the *rr07* overexpressed WT-*strHgfp*-*rr07*over strain (Fig. S5A), verifying the successful construction of a strain highly expressing the N-glycan degradation genes. Next, by inoculating the same amounts of cells into BHI broth, a coculture of the wild-type and *rr07*over (wild-type strain with *rr07* overexpressing) strain was established, and the respective monocultures were included in parallel. Each culture was subcultured for three consecutive transfers, and the numbers of colony forming units (CFU) of the wild-type and the *rr07*over strain were counted from each subculture. It turned out that the *rr07*over strain had 20% fewer cells than did the wild-type strain in the first generation of coculture, and this further decreased to 40% fewer in the third generation of the coculture (Fig. S5B). This determined that the induction of the N-glycan degradation genes reduces the intraspecies competitiveness of pneumococcus, while in contrast, the lower expression of the N-glycan degradation genes in the presence of glucose confers a fitness advantage to this bacterium.

## DISCUSSION

**FIG 6:**
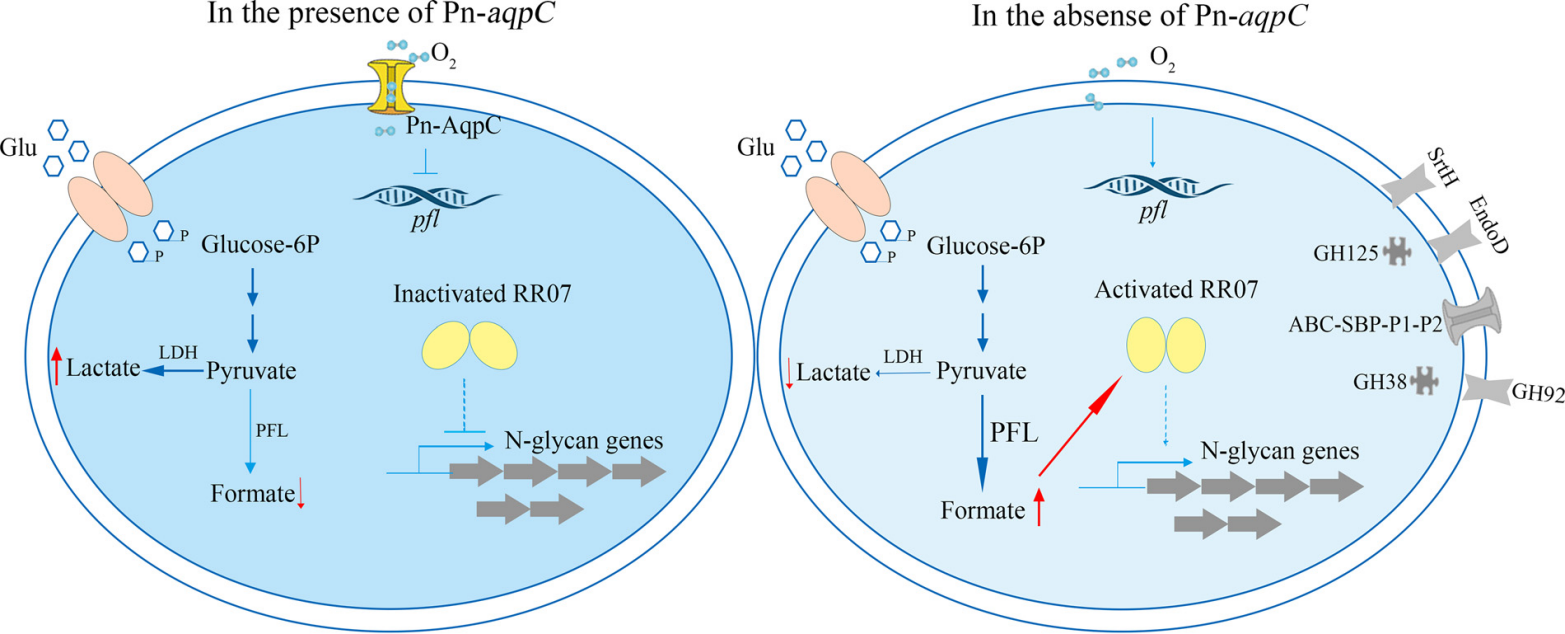
Pn-AqpC-mediated fermentation pattern coordinating with the two-component system 07 regulates host N-glycan degradation of Streptococcus pneumoniae. In the presence of Pn-AqpC and higher levels of cellular O_2_ (left), glucose is homogenously fermented as lactate, and RR07 is maintained as inactive so that the N-glycan degradation genes are repressed. While in the absence of Pn-AqpC (right), the *pfl* gene is induced, and formate production is elevated. Formate acts as an activator to activate RR07 and thus induces the N-glycan degradation genes.

As a human opportunistic pathogen, Streptococcus pneumoniae is capable of utilizing complex N-glycans on human cell surfaces, which is supported by a complete suite of the N-glycan degradation genes in the pneumococcal genome (19, 20). However, how these N-glycan degradation genes are regulated and the physiological impacts of the expression of these genes are poorly understood in pneumococcus. In this study, through proteomics and experimental verification, we found a remarkably higher expression of N-glycan degradation genes in pneumococcus R6 lacking the aquaporin Pn-AqpC, which facilitates O_2_ permeation into cells. Meanwhile, the deletion of Pn-*aqpC* also induced the expression of the pyruvate formate lyase and increased the production of formate. Furthermore, the two-component system regulator RR07 was determined to be involved in the regulation of N-glycan degradation gene induction due to the deletion of Pn-*aqpC*. However, RR07 activation occurred through a phosphorylation-independent mode. Based on the increased formate production in the absence of Pn-AqpC, we further determined formate to be a newly identified activator of RR07. Therefore, in the presence of Pn-AqpC, pneumococcus maintains a microaerobic cellular environment, and performs the homolactic acid fermentation of glucose while in the absence of Pn-AqpC, pyruvate formate lyase is activated, and the metabolite formate activates RR07, in turn inducing the N-glycan degradation genes (Fig. 6). However, the overexpression of the N-glycan degradation genes is disadvantageous to pneumococcus. Therefore, controlled N-glycan degradation is important for pneumococcus in the trade-off between fitness and pathogenesis.

As an opportunistic pathogen, successfully colonizing the human upper respiratory tract is the first step for pneumococcus to become infectious (29), and the metabolism of the N-glycans attached to glycoproteins is critical for pneumococcus at this phase, as N-glycan utilization not only provides a carbon source for pneumococcus but also exposes receptors on host cell surfaces to promote pneumococcal attachment and invasion (12). The recently identified N-glycan degradation genes of pneumococcus are sparsely distributed in the genome (18, 20). Recently, it has been reported that the two-component-system TCS07 of pneumococcus D39 activates the expression of N-glycan degradation genes when growing in fetuin (20). However, the specific ligand for TCS07 activation remains unknown. Unexpectedly, we found that formate, the anaerobic metabolite of glucose fermentation, but not HK07-relayed phosphorylation, could activate the response regulator RR07 of TCS07, and subsequently induced N-glycan degradation genes (Fig. 4 and 5; Fig. S3). This is different from the classical activation mode of the response regulator (RR) of a two-component system via a phosphorylation relay from its cognate histidine kinase (HK) (30–32). Formate has also been reported as an activator of other transcription regulators, such as CcpA and GlnR, presumably through altering protein conformations (28). Distinct from the other 12 pneumococcal RRs (30), RR07 belongs to the AraC/XylS transcription factor family, and there is a unique, large insertion of approximately 231 amino acids (linker region) between the phosphor-receiving domain and the DNA binding domain (25) (Fig. S6). This linker shows no evident similarity to other known protein sequences in the PDB database. Thus, no known functions can be predicted. It has been speculated that RR07 can be activated via dual control modes: a phosphorylation relay from the cognate histidine kinase and noncovalent ligands acting at the linker region (25). In this study, a LC-MS/MS analysis did not detect formylation modification, which can be formed by the covalent linking of formic acid to Ser, Thr, or Lys residues and can regulate protein functions in eukaryotes (33, 34), in the RR07-6×His protein immunoprecipitated from both the wild-type and ΔPn-*aqpC* strains (Data Set S1), supporting the claim that formate acts as a noncovalent ligand. As electrophoretic mobility shift assay (EMSA) assay did not detect the binding of RR07 to the promoter regions of *strH* and *gh125* (Fig. S7), whether formate functions as a ligand to modulate RR07 activity needs to be verified when the regulatory pathways of RR07 on N-glycan degrading genes are identified.

However, enhanced N-glycan degradation may not be beneficial to pneumococcus (as indicated in Fig. S5B), though the utilization of human N-glycans promotes the infection of pneumococcus. Hence, controlled N-glycan degradation could be a trade-off strategy for pneumococcus to modulate between invasiveness and its own growth fitness. We did find that in the presence of glucose, the pneumococcal N-glycan degrading genes appeared to be repressed, as almost no expression of these genes was observed in the wild-type strain R6 (Fig. 2). Moreover, we further demonstrated that pneumococcal N-glycan metabolism was also subject to carbon catabolite repression (CCR), a regulatory mechanism employed by bacteria to utilize preferred carbon sources (in general, glucose) over nonpreferred ones when more than one kind of carbohydrate is present (35, 36). We found a 10.4-fold higher expression of *strH* in 2% fetuin and a 3.4-fold decrease by the addition of 0.5% glucose in 2% fetuin (Fig. S5C). This suggests that in addition to formate-RR07 regulation, CCR is also involved in the regulation of N-glycan metabolism by pneumococcus. In the model Gram-positive bacterium Bacillus subtilis, carbon catabolite repressor protein A (CcpA) is a central regulator of CCR (37). However, in pneumococcus, CcpA has been only found in the CCR of lactose-inducible beta-galactosidase activity (13). Most CCR mechanisms that control the utilization of other carbohydrates in pneumococcus are independent of CcpA, while the histidine-containing phosphocarrier protein (Hpr) coordinates with pathway specific transcriptional regulators to control the CCR of pneumococcal galactose metabolism (38). Whether CcpA or Hpr is also involved in the CCR regulation of pneumococcal N-glycan metabolism needs to be clarified.

Pneumococcus routinely performs glucose homolactic acid fermentation via lactate dehydrogenase (LDH) (15), while in the absence of the oxygen facilitator Pn-AqpC, the *pfl* gene is upregulated, meaning that PFL catalyzes more formate production, which leads to the activation of RR07 and the consistent upregulation of the expression of N-glycan degrading genes (Fig. 1 and 4). Homolactic acid fermentation is also important for pneumococcal virulence, as the deletion of the *ldh* gene significantly attenuates the virulence of blood infected pneumococcus (39). It is believed that the homolactic acid fermentation of pneumococcus is controlled by two factors regulating PFL expression: one is CcpA, the key regulator controlling the carbon flux to lactate and suppressing the *pfl* gene through directly binding to the *pfl* promoter (28, 40), while the other is the Embden-Meyerhof-Parnas (EMP) pathway intermediates, dihydroxyacetone-phosphate (DHAP) and fructose-1,6-bisphosphate (FBP), which repress and activate PFL and LDH, respectively (41, 42). The deletion of Pn-*aqpC* induces both the *pfl* and *adh* genes (Fig. 1A and E; Data Set S1), thereby shifting glucose catabolism from homo-acid to mixed acid fermentation and increasing formate production. However, the deletion of Pn-*aqpC* did not change the expressions of *ccpA* and the amounts of intracellular DHAP and FBP (data not shown). This suggests that Pn-AqpC could be a third factor in the regulation of *pfl* and glucose fermentation by controlling the cellular oxygen content.

In conclusion, this work revealed that in the presence of the oxygen facilitator Pn-AqpC, pneumococcus performs homolactic acid fermentation from glucose and maintains *pfl* repression, while in the absence of Pn-AqpC, the *pfl* gene is induced, which increases formate production. Formate, as a newly identified activator, activates RR07, the response regulator of TCS07, and subsequently induces the host N-glycan degradation genes of pneumococcus. However, excessive N-glycan degradation can decrease the fitness of pneumococcus, and regulated N-glycan degradation is a trade-off strategy used by pneumococcus to balance between maintaining fitness and attacking hosts. Therefore, this highlights the significance of the fermentation pattern coordinated TCS07 regulation mechanism on the N-glycan degradation reported in this work. In addition, CCR also appears to be involved in the regulation of N-glycan degradation by pneumococcus.

## MATERIALS AND METHODS

### Experimental strains and culture conditions.

The bacterial strains used in this study are listed in Table S1. Pneumococcal strain R6 (22) and its derivates were grown in brain heart infusion (BHI, Difco, BD) broth or on a BHI agar plate supplemented with 3% (vol/vol) defibrillated sheep blood at 37°C with 5% CO_2_. For the test of pneumococcal growth in glucose or N-glycan, R6 was grown in C+Y medium (43), omitting glucose and sucrose (sugar-omit C+Y) with supplementation of 0.5% glucose or 2% fetuin or 0.5% glucose plus 2% fetuin. Escherichia coli strains were grown in Luria-Bertani (LB) medium at 37°C under shaking at 200 rpm/min. For the transformants selection, spectinomycin (200 and 50 μg/mL), kanamycin (250 and 50 μg/mL), chloramphenicol (5 and 34 μg/mL), or gentamicin (50 and 40 μg/mL) were used for pneumococcus and E. coli, respectively.

### Construction of mutation strains.

All of the primers used in this study are listed in Table S2. The Δ*rr07*, Δ*hk07*, ΔPn-*aqpC*/*rr07*, and ΔPn-*aqpC*/*hk07* strains were constructed using PCR ligation methods (44). Briefly, the DNA fragments upstream and downstream of the *rr07* or *hk07* genes, approximately 1,000 bp in length, were PCR amplified, respectively, using pairs of primers listed in Table S2. After purification, the PCR products were digested with NheI and ligated with the NheI-cut spectinomycin resistance gene fragment, which was PCR amplified from the shuttle plasmid pDL278 (45) using a pair of primer integrating NheI recognizing sequences at a mole ratio of 1:1:1, using T4 DNA ligase. Then, the ligation mixtures were transformed into the S. pneumoniae R6 and ΔPn-*aqpC* strains as described previously (46). The correct transformants were selected on BHI blood agar plates supplemented with spectinomycin or spectinomycin and kanamycin and then verified by PCR amplification and sequencing.

For construction of the *rr07* complementary strain, the promoter fragment of a three-gene operon comprised of spr0152, *hk07*, and *rr07* was PCR amplified, purified, and fused with a PCR amplified *rr07* gene fragment via overlapping PCR to obtain the Pro-*rr07* fragment. The Pro-*rr07* fragment was purified and integrated into shuttle plasmid pIB166 (47) using a Gibson assembly cloning kit (NEB, Beverly, MA). The constructed plasmid pIB166Pro-*rr07* was transformed into R6 wild-type, WT-*strHgfp*, and ΔPn-*aqpC/rr07*-*strHgfp* strains to construct the *rr07*over, WT-*strHgfp-rr07*over, and ΔPn-*aqpC/rr07*-*strHgfp*com*rr07* strains. For the construction of the *rr07*D55A complementary strain, the Asp at the 55^th^ position of RR07 was mutated into alanine on the pIB166Pro-*rr07* and then transformed into the ΔPn-*aqpC/rr07*-*strHgfp* strain to get the *aqpC/rr07*-*strHgfp*com*rr07*D55A strain. The correct transformants were verified by PCR amplification and sequencing.

### Construction of reporter strains and His tagged strains.

The stop codon-lacking DNA fragments of the *strH* gene, encoding beta-N-acetylhexosaminidase, and *gh125* gene, encoding alpha-1,6-mannosidase, were PCR amplified with their inherent promoter, using genome DNA as the template. Meanwhile, a DNA fragment of superfolding green fluorescence protein gene (sf-*gfp*) was amplified from plasmid pYES2-*So*-*aqpA*-*gfp* (48) and inserted into pneumococcal intergrative plasmid pPEPX1, which was constructed by substituting a spectinomycin resistance gene on pPEPX-Plac (49) (Addgene, number 122632) with a gentamicin resistance gene fragment at the BglII and BamHI sites. Then, the *strH* and *gh125* DNA fragments were integrated into pPEPX1-*gfp* at the EcoRI and BglII sites. The constructed plasmid pPEPX1-*strHgfp* or pPEPX1-*gh125gfp* was transformed into the R6 wild-type and ΔPn-*aqpC* strains. The integration of plasmids pPEPX1-*strHgfp* and pPEPX1-*gh125gfp* into the pneumococcal genome would be either at the *strH* or the *gh125* gene location via single-crossover homologous recombination or at genome location between spr1700 (homologue of SPV_1665) and spr1701 (homologue of SPV_2404) via double-crossover homologous recombination. The correct transformants with the same genetic background were verified by PCR amplification and sequencing.

For the construction of the *rr07*-6XHistidine tagged strain, a DNA fragment of the *rr07* gene, at a length of approximately 1,000 bp, was PCR amplified using a pair of primers with a reverse primer carrying 6 histidine encoding sequences immediately before the stop codon. Then, the DNA sequences immediately downstream of the *rr07* gene were also PCR amplified. The PCR products were purified and digested with BamHI and ligated with a BamHI-digested spectinomycin resistance gene fragment at a mole ratio of 1:1:1 using T4 DNA ligase. Then, the ligated mixture was transformed into the R6 wild-type strain and the ΔPn-*aqpC* mutant. The correct WT-*rr07*-6×His and ΔPn-*aqpC*-*rr07*-6×His strains were verified by PCR amplification and sequencing.

### TMT proteomics analysis.

The pneumococcus R6 wild-type and ΔPn-*aqpC* strains were grown in BHI broth in triplicate. Mid-exponential cells were collected, washed twice with PBS, and broken by sonication for 30 min. After centrifugation at 12,000 rpm for 15 min, the cell lysates were collected, and the protein concentrations were measured using a bicinchoninic acid (BCA) protein assay kit (Thermo Fisher Scientific, Waltham, MA). The protein samples were reduced with 5 mM dithiothreitol for 30 min at 56°C and alkylated with 11 mM iodoacetamide for 15 min at room temperature in darkness. Then, the proteins were digested with trypsin overnight, and the digestion-produced peptides were dissolved in 0.5 M triethylammonium hydrogen carbonate (TEAB) and processed according to the manufacturer’s protocol for the TMT kit (Thermo Fisher Scientific). Briefly, one unit of TMT reagent was thawed and reconstituted in acetonitrile. The peptide mixtures were incubated with the TMT reagent for 2 h at room temperature and then pooled, desalted, and dried via vacuum centrifugation. The peptides were fractionated by high pH reverse-phase HPLC using an Agilent 300Extend C18 column (5 μm particles, 4.6 mm ID, 250 mm length). Then, the tryptic peptides were separated using a 65-min gradient elution at a flow rate of 400 nL/min on an EASY-nLC 1000 UPLC system and subjected to an NSI source, followed by tandem mass spectrometry (MS/MS) in a Q Exactive Plus (Thermo Fisher Scientific) coupled online to the UPLC. The electrospray voltage applied was 2.0 kV. The *m/z* scan range was 350 to 1,800 for the full scan, and intact peptides were detected in the Orbitrap at a resolution of 70,000. Peptides were then selected for MS/MS using an NCE setting of 28, and fragments were detected in the Orbitrap at a resolution of 17,500. The data-dependent procedure alternated between one MS scan followed by 20 MS/MS scans. The resulting MS/MS data were processed using the Maxquant search engine (v.1.5.2.8). Tandem mass spectra were searched against a Streptococcus pneumoniae R6 uniprot database concatenated with a reverse decoy database. Trypsin was specified as the cleavage enzyme, allowing up to 2 missing cleavages. The mass tolerance for precursor ions was set as 20 ppm in the First Search and 5 ppm in the Main Search, and the mass tolerance for the fragment ions was set to 0.02 Da. The carbamidomethyl on Cys was specified as a fixed modification, and the acetylation modification and oxidation on Met were specified as variable modifications. The false discovery rate (FDR) was adjusted to <1%, and the minimum score for modified peptides was set to >40.

### Clusters of Orthologous Groups (COG) and Kyoto Encyclopedia of Genes and Genomes (KEGG) pathway annotation and enrichment analysis.

The differentially expressed proteins were BLASTed against the COG database to define the functional classifications and against KEGG databases to analyze the pathways in which the proteins are involved. The KEGG Pathway database was used to identify enriched pathways by a two-tailed Fisher’s exact test to test the enrichment of the differentially expressed proteins against all identified proteins. A pathway with a corrected *P* value of <0.05 was considered statistically significant.

### GFP fluorescence determination.

The GFP reporter cells were collected, washed twice, and resuspended in PBS. After exposure to air for 30 min in the dark, 200 μL of cells were added into a 96-well plate, and the GFP fluorescence intensities were measured using a Synergy H4 hybrid microplate reader (BioTek) at an excitation wavelength of 480 nm and an emission wavelength of 520 nm. Meanwhile, 40 μL of PBS-resuspended cells were placed on a polysine microscope slide (25 × 75 × 1mm) (Thermo Fisher Scientific, Waltham, MA), covered with a Fisherbrand microscope cover glass slip (15 mm diameter, 0.13 to 0.17 mm thick) (Thermo Fisher Scientific), and visualized under a confocal laser scanning microscope (Leica TCS SP8, Leica Microsystems, Buffalo Grove, IL, USA). Excitation was provided at 488 nm, and emission was collected from a range of 500 to 600 nm.

### High pressure liquid chromatography (HPLC) measurement of fermentation metabolites.

The pneumococcal R6 wild-type and ΔPn-*aqpC* strains were grown in BHI broth until the early stationary-phase, and then the cultures were centrifuged at 12,000 rpm for 15 min. The supernatants were filtered using a 0.22 μm disposable needle filter (Millipore, Billerica, MA).Then, the formate, acetate, and lactate concentrations in the supernatants were measured using HPLC (Thermo Fisher Scientific). The chemicals formate, acetate, and lactate at known concentrations were included and the concentrations of corresponding chemicals in the tested samples were calculated according to chemical standards.

### Quantitative reverse transcription-PCR (qRT-PCR).

Total RNA was extracted from mid-exponential (OD_600_ value of approximately 0.4) pneumococcal cells using the TRIzol reagent (Invitrogen, Carlsbad, CA) as recommended by the suppliers. After quality confirmation with a 1% agarose gel, the RNA was treated with RNase-free DNase (Promega, Madison, WI) and analyzed by PCR for possible chromosomal DNA contamination. cDNA was generated from 2 μg total RNA with random primers using Moloney murine leukemia virus reverse transcriptase (Promega, Madison, WI), according to the supplier’s instructions. The cDNA was used for quantitative-PCR (qPCR) amplification with the corresponding primers (Table S2). Amplifications were performed with a Mastercycler ep realplex^2^ (Eppendorf, Germany). To estimate the copy numbers of tested genes, a standard curve of each tested gene was generated via qPCR, using the 10-fold serially diluted PCR product as the template. The 16S rRNA gene was used as the biomass reference. The number of copies of tested gene transcript per 1,000 16S rRNA copies is shown (Fig. 2C and D, 3A, 4A; Fig. S1, S2). All of the measurements were done for triplicate samples and were repeated at least three times.

### Immunoprecipitation (IP), liquid chromatography-tandem mass spectrometry (LC-MS/MS) identification and phosphorylation determination of the RR07 protein.

The 6×His tagged RR07 protein was purified via IP using anti-His-tag MAb-magnetic agarose (MBL International Corporation, Woburn, MA) according to the instructions of the manufacturer. Briefly, the 6×His-tagged-RR07 expressing strains WT-*rr07*-6×His and ΔPn-*aqpC*-*rr07*-6×His were statically grown in BHI broth. The mid-exponential cells were collected and washed with PBS three times. Then, the cells were resuspended in lysis buffer (50 mM Tris-HCl, 150 mM NaCl, 0.05% NP-40, 1 mM DTT), and sonicated on ice for 30 min. After centrifugation at 8,000 rpm for 10 min, the supernatant was mixed and incubated with magnetic beads. After being washed 4 times with lysis buffer, the immunoprecipitated 6×His-tagged RR07 protein was eluted by boiling in nonreducing SDS sample buffer (4% SDS, 125 mM Tris-HCl [pH 8.0], 20% glycerol) and separated using 15% SDS-PAGE. The target RR07 protein band with the expected molecular size was sliced and cut into pieces. The gel pieces were reduced with 5 mM dithiothreitol, alkylated with 55 mM iodoacetamide at 37°C for 1 h, and then digested using trypsin (Promega, Fitchburg, WI) at 37°C overnight. The digested products were separated using a 65 min gradient elution at a flow rate of 0.250 mL/min with the EASY-nLC integrated nano-HPLC system (Thermo Fisher Scientific), which was directly interfaced with a Thermo Q-Extractive mass spectrometer. The Q-Extractive mass spectrometer was operated in the data-dependent acquisition mode and used the Xcalibur 3.0 software package. A single full-scan mass spectrum in the Orbitrap (350 to 1,500 *m/z*, 120,000 resolution) was followed by 20 data-dependent MS/MS scans in the ion trap at 27% normalized collision energy. Each mass spectrum was searched against the RR07 protein sequence (Uniprot accession number Q8DRF5) using the SEQUEST search engine of the Proteome Discoverer software package (v2.4). Trypsin was specified as the cleavage enzyme, allowing up to 2 missing cleavages. The mass tolerance for precursor ions was set to 20 ppm, and the mass tolerance for fragment ions was set to 0.02 Da. The carbamidomethyl on Cys was specified as a fixed modification, whereas the oxidation on Met, the formylation on Lys, Ser and Thr, and the phosphorylation on Asp, Ser, and Thr were specified as variable modifications. The false discovery rate was adjusted to <1%.

The phosphorylation status of IP pulldown RR07-6×His proteins was determined with the Pro-Q Diamond Phosphoprotein Gel Stain (Thermo Fisher Scientific) and Phos-tag Acrylamide gel (Whatman) per the protocols supplied by the commercial suppliers.

### Overexpression of the RR07-6×His protein.

A 1,287 bp DNA fragment containing the entire *rr07* coding region was PCR amplified with the primers listed in Table S2 and then integrated into pET-28a (Novagen, Madison, WI) via Gibson assembly (NEB) to produce pET-28a-*rr07*. The verified constructs were transformed into E. coli BL21(DE3) (Novagen). The positive transformants were grown at 37°C to an OD_600_ of 0.6 to 0.8, and 0.1 mM IPTG (Sigma-Aldrich, St. Louis, MO, USA) was added to the cultures to induce protein expression. Then, the cultures were further incubated at 37°C for 4 h. Next, the cells were collected by centrifugation, resuspended in binding buffer (20 mM Tris-HCl, 500 mM NaCl, 20 mM imidazole and 1 mM DTT, pH 7.4), and lysed by sonication for 45 min. The supernatant was filtered and applied to a Ni^2+^-charged chelating column (GE Healthcare, Piscataway, NJ) that was previously equilibrated with binding buffer. The proteins were then eluted using elution buffer (20 mM Tris-HCl, 500 mM NaCl, 500 mM imidazole, and 1 mM DTT, pH 7.4). The fractions with desired proteins were pooled and dialyzed against buffer containing 20 mM Tris-HCl, 150 mM NaCl and 1 mM DTT three times. The purified RR07-6×His protein was then stored in aliquots in 10% glycerol at −80°C until use.

### Statistical analysis.

A one-way analysis of variance (ANOVA) followed by Tukey’s *post hoc* test and Student's *t* test were performed using PASW Statistics 18 or Excel, respectively.
