# Supplementary material for: Pn-AqpC-Mediated Fermentation Pattern Coordination with the Two-Component System 07 Regulates Host N-Glycan Degradation of Streptococcus pneumoniae
Source: Microbiol Spectr. 2022 Sep 15;10(5):e02496-22. doi: 10.1128/spectrum.02496-22 (PMC9603416; doi:10.1128/spectrum.02496-22)
Supplement: Supplemental file 1 — Fig. S1 to S7 and Tables S1 and S2. Download spectrum.02496-22-s0001.pdf, PDF file, 0.8 MB [file spectrum.02496-22-s0001.pdf]

Supplementary Information for

Pn-AqpC-mediated fermentation pattern coordination with the two-component system 07 regulates host N-glycan degradation of *Streptococcus pneumoniae*

Kaiqiang Shen[a,b,1], Qingqing Hu[a,1], Lin Zhu[a,1], Wenshuang Miu[c], Yuzhu Dong[a], Fu Ren[c], Xiuzhu Dong[a,b,*], Huichun Tong[a,b,*]

[a]State Key Laboratory of Microbial Resources, Institute of Microbiology, Chinese Academy of Sciences, Beijing 100101, China

[b]University of Chinese Academy of Sciences, Beijing 100049, China

[c]School of Basic Medicine, Shenyang Medical College, Shenyang 110000, China

This file contains:

Supplementary Figures S1-S7

Supplementary Tables S1-S2

Other supplementary material for this manuscript include the following:

Dataset S1 under captions: Sheet 1. Proteins identified by TMT quantitative proteomic in pneumococcus R6 wild-type and Pn-*aqpC* deletion strain; Sheet 2. TMT quantitative proteomic analysis identifies differentially expressed proteins in Pn-*aqpC* deletion strain. Sheet 3. LC-MS/MS determines the variable modifications of RR07 protein immuno-precipitated from wild-type strain. Sheet 4. LC-MS/MS determines the variable modifications of RR07 protein immuno-precipitated from Pn-*aqpC* deletion strain

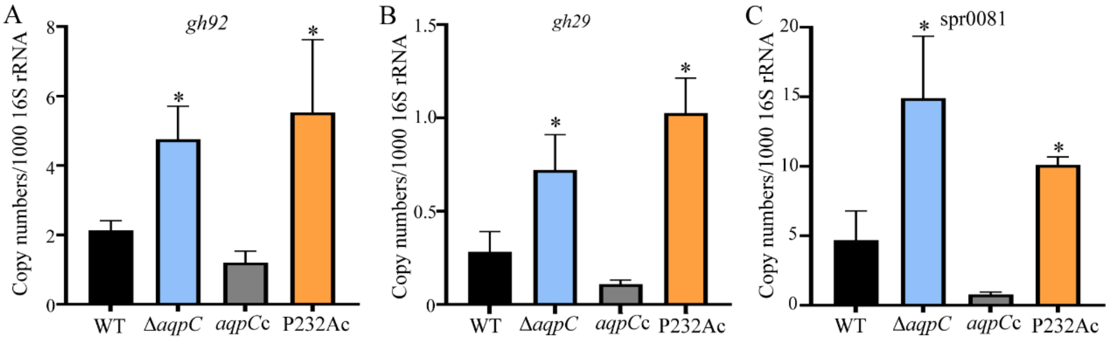

Fig. S1. Deletion of Pn-*aqpC* increases transcription of N-glycan degradation genes in pneumococcus R6. The wild-type (WT), ΔPn-*aqpC* (Δ*aqpC*), Pn-*aqpC* complementary (*aqpC*c) and P232A mutated Pn-*aqpC* complementary (P232Ac) strains were grown in BHI broth, and mid-exponential cells were collected for total RNA extraction. qRT-PCR was implemented to measure the transcription levels of *gh92* (A), *gh29* (B) and spr0081 (C). The results are expressed as transcript copy numbers per 1000 16S rRNA. For A, B and C, the experiments were repeated for three times with triplicate samples for each experiment. Averages ± SD from three independent experiments are shown. *, significantly different from the datum of the wild-type strain; One way ANOVA; Tukey's post hoc test ($P < 0.05$).

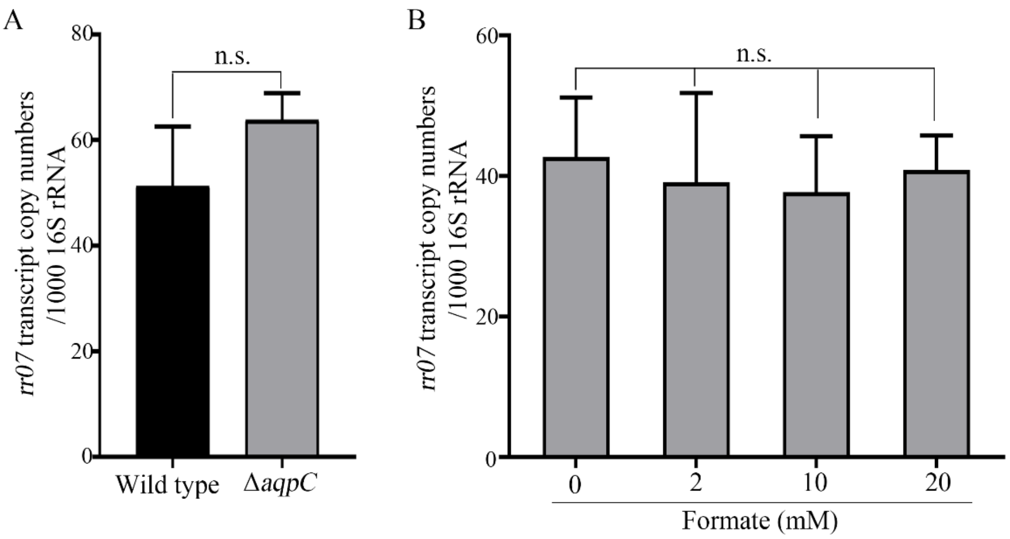

Fig. S2. qRT-PCR determines the transcript copies of *rr07* gene in pneumococcus R6. A. The wild-type and ΔPn-*aqpC* (Δ*aqpC*) strains were grown in BHI broth and mid-exponential cells were collected for total RNA extraction. B. The wild-type strain was grown in BHI broth without or with supplementation of 2, 10 and 20 mM formate, mid-exponential cells were collected for total RNA extraction. qRT-PCR was implemented to quantify the transcript copies of the *rr07* gene. The results are expressed as transcript copy numbers per 1000 16S rRNA. The experiments were repeated for three times, with triplicate samples included for each experiment. Averages ± SD of three independent experiments are shown. n.s., no significant difference is identified between the strains by Student's *t* test (A) and One way ANOVA; Tukey's post hoc test (B) ($P > 0.05$).

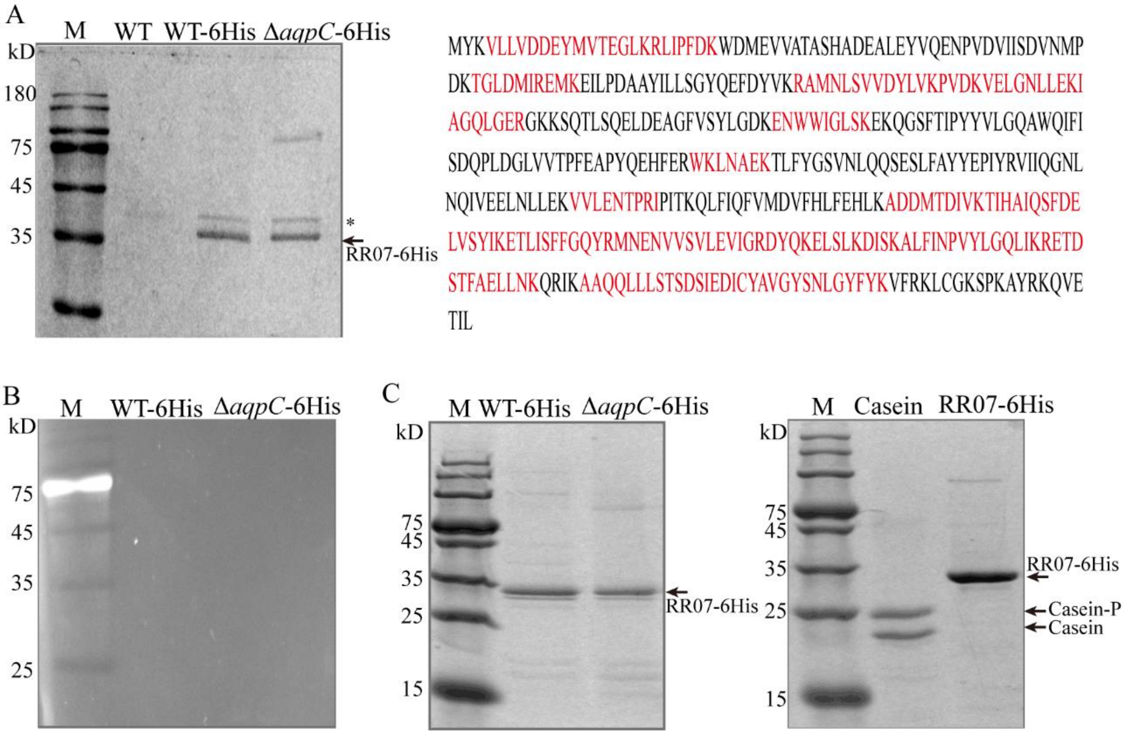

Fig. S3. Determination of RR07 protein phosphorylation in pneumococcus R6 and Pn-*aqpC* deletion mutant. A. Immuno-precipitation of RR07-6His protein from the wild-type and ΔPn-*aqpC* strains. *rr07*-6His strain was constructed by fusing the 6XHis encoding sequence to the C-terminus of *rr07* at the genomic locus of the wild-type strain and ΔPn-*aqpC* mutant. Mid-exponential WT-*rr07*-6His (WT-6His) and ΔPn-*aqpC*-*rr07*-6His (Δ*aqpC*-6His) cells were collected, and cellular RR07-6His proteins were immune-precipitated from the cell lysates and examined on 12% SDS-PAGE gel (left panel). The wild-type strain (WT) was included as a negative control. The protein band of assumed RR07 specified by black arrow was subjected to LC-MS/MS analysis, and the identified peptide fragments (with amino acid sequences in red letters) are shown in the right panel. B and C. Assays of the phosphorylation status of RR07-6His proteins. IP-pulled down RR07-6His proteins from WT-*rr07*-6His (WT-6His) and ΔPn-

aqpC-rr07-6His (Δ*aqpC*-6His) strains were run on 12% SDS-PAGE gel and then stained with Pro-Q™ Diamond Phosphoprotein Gel Stain (B) and Phos-tag™ Acrylamide gel (C left panel). The phosphorylated protein Casein was included as a positive control and recombinant RR07-6His protein was also examined for Phos-tag™ Acrylamide gel (C right panel). M, protein molecular markers.

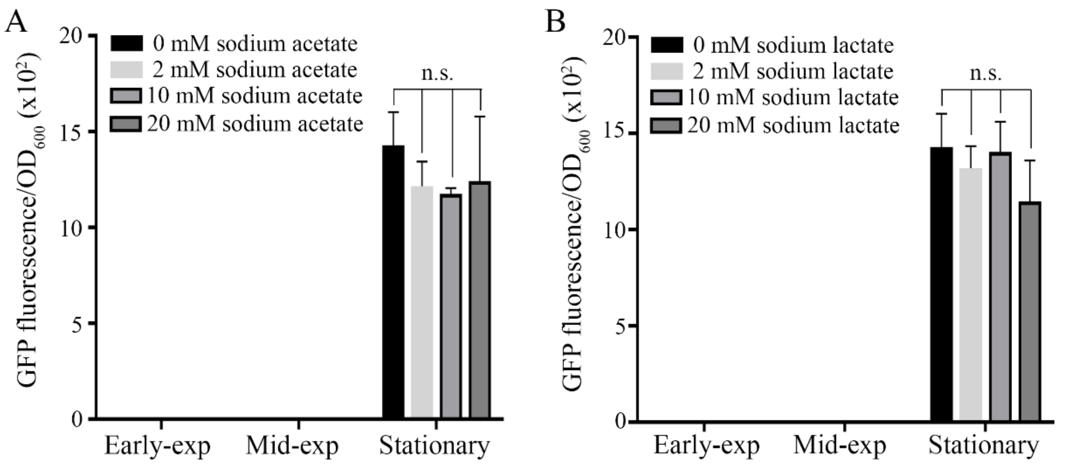

Fig. S4. Determination of acetate and lactate induction of N-glycan degradation gene *strH* in pneumococcus strain R6. The WT-*strHgfp* strain was grown in BHI broth with or without supplementation of gradient concentrations of sodium acetate (A) and sodium lactate (B). The optical density at 600 nm (OD_{600}) and intensities of GFP fluorescence were measured for early-, mid-exponential and stationary cells. The results are expressed as GFP fluorescence/OD_{600}. The experiments were repeated for three times with triplicate samples for each experiment. Averages ± SD from three independent experiments are shown. n.s., no significant difference is identified between the strains by one way ANOVA analysis, Tukey's post hoc test ($P > 0.05$).

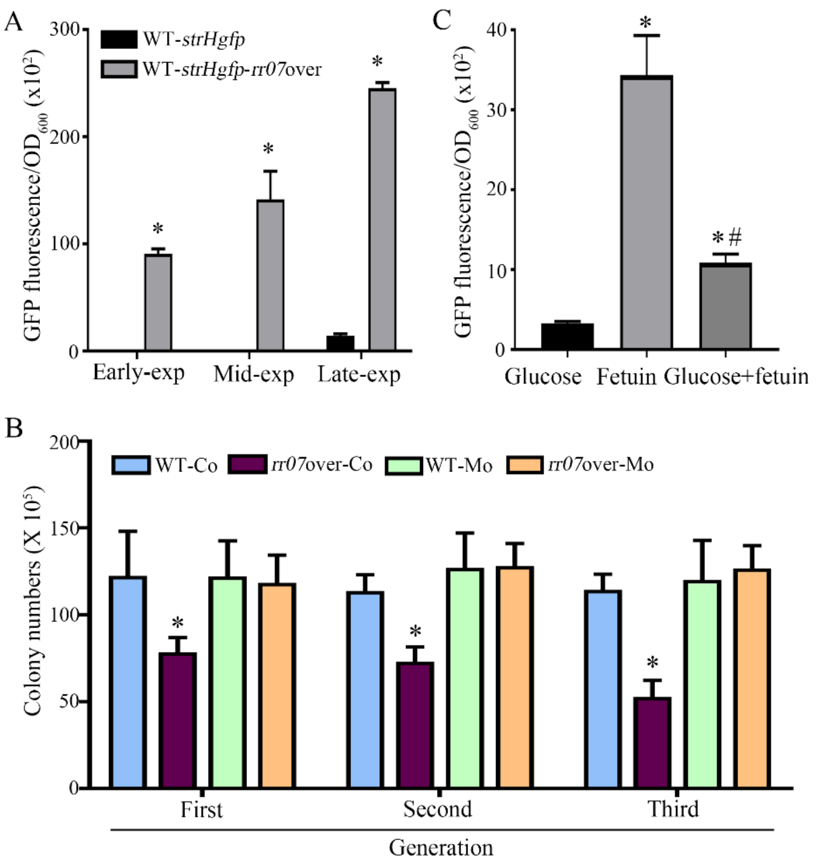

Fig. S5. Induced expression of N-glycan degradation gene reduces the intra-species competitiveness of pneumococcus R6 and N-glycan degradation genes are subject to CCR control in R6. A. Overexpression of *rr07* elevated StrH expression in R6. *rr07* gene was cloned into plasmid pIB166 and then transferred into WT-*strHgfp* to construct the WT-*strHgfp-rr07*over strain. The WT-*strHgfp-rr07*over and WT-*strHgfp* strains were grown in BHI broth and optical density at 600 nm (OD_{600}) and GFP fluorescence were measured for early-, mid- and late-exponential (exp) cells. The results are expressed as GFP fluorescence/OD_{600}. *, significantly different from the datum of WT-*strHgfp* strain at respective growth phase. Student's *t* test, $P < 0.05$. B. Competition between the wild-typ (WT) and *rr07* overexpressed wild-type (*rr07*over) strains was determined for three successive sub-cultures. The same amounts of the two strains were

co-inoculated (Co) or mono-inoculated (Mo) in BHI broth, and the co-culture and mono-culture were sub-cultured for three successive generations. Numbers of Colony forming units (CFUs) in each generation were counted on BHI agar plate with and without supplementation of spectinomycin; CFUs of the *rr07*over strain in co-cultures were those on the spectinomycin-containing BHI agar, and the wild-type strain CFUs were the difference of CFUs on spectinomycin-void and -added BHI agars. *, significantly different from the data of other strains in respective generation as verified by one way ANOVA analysis followed by Tukey's post hoc test ($P < 0.05$). C. The overnight culture of WT-*strHgfp* strain was 1:30 diluted into fresh sugar-omitted C+Y medium with extra supplementation of 0.5% glucose (Glucose), or 2% fetuin (Fetuin), or 0.5% glucose plus 2% fetuin (Glucose+fetuin). Late exponential cells were measured for optical density at 600 nm (OD_{600}) and GFP fluorescence. The results are expressed as GFP fluorescence/OD_{600}. For A-C, the experiments were repeated for three times with triplicate samples for each experiment. Averages ± SD of three independent experiments are shown. * and $^{\#}$, significantly different from the datum in glucose and fetuin, respectively. One way ANOVA, Tukey's post hoc test ($P < 0.05$).

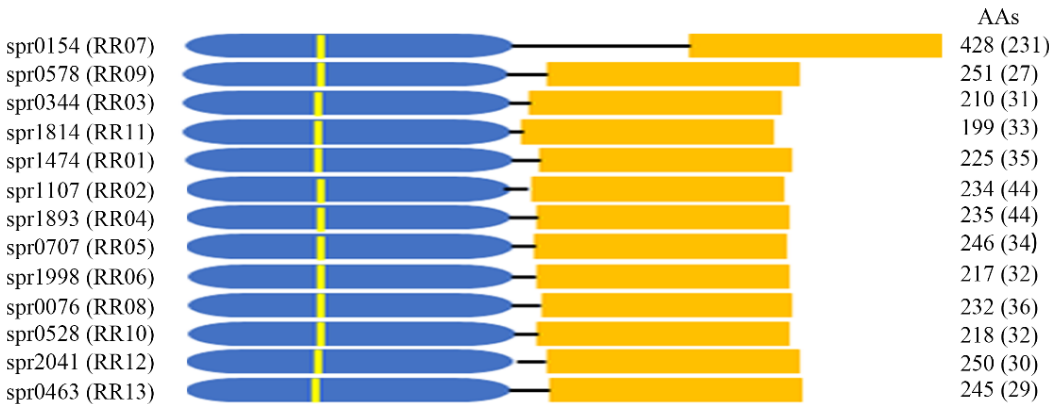

Fig. S6. Motif analysis of the response regulators of 13 two-component-systems in pneumococcus R6. Amino acid sequences of the response regulators (RR) were retrieved from the KEGG database, and the protein motifs were analyzed using the Pfam database. Blue ovals represent the phosphate receiver domains, and the yellow vertical lines specify the aspartic acid residue receiving the phosphoryl group from the histidine kinase. Yellow bars represent the HTH-helix DNA-binding domains, and the black lines between the two represent the linker regions between the phosphate receiver domain and DNA-binding domain. Gene accession numbers (with the identifiers in the parenthesis) of 13 response regulators, and the amino acids (AAs) numbers of the whole length response regulators (with linker region in the parenthesis) are shown at the left and right, respectively.

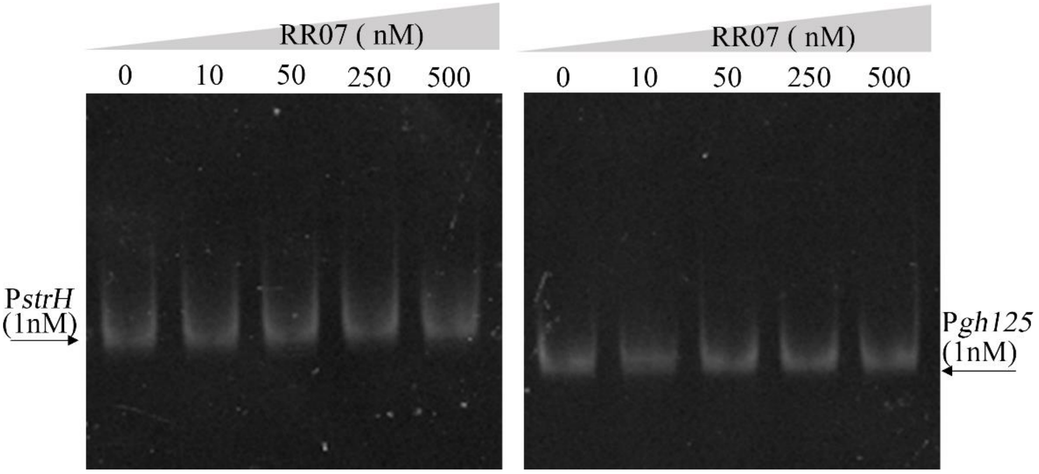

Fig. S7. Electrophoretic mobility shift assay (EMSA) assays the binding of RR07 to the promoter regions of *strH* and *gh125* genes. Promoter DNA fragments of *strH* (P*strH*, left panel) and *gh125* (P*gh125*, right panel) were PCR amplified using primers listed in Table S2. One nanomolar of dsDNA probe and increasing amounts of RR07 protein (labelled on the top) were mixed in the binding buffer (20 mM Tris-Cl, pH 8.0, 10% glycerol, 1 mM EDTA, 1 mM DTT and 50 mM NaCl). The reactions were stayed at 30°C for 30 min, and then was electrophosized on 6% polyacrylamide gel on ice. The gel was stained with 1:10000 diluted SYBR Gold Nucleic Acid Gel Stain (Invitrogen) (1) for 30 min, and photographed under ultraviolet transillumination using Bio-Rad GelDoc XR.

Table S1. Strains and plasmids used in this study[a]

Strains and plasmids	Description	Reference
**Strains**		
**pneumococcal strains**		
R6	Wild-type strain, Spec^{s}, Kan^{s}	2
ΔPn-*aqpC*	R6 strain with Pn-*aqpC* deletion, Kan^{r}	2
Pn-*aqpC*com	ΔPn-*aqpC* strain with Pn-*aqpC* ectopically complemented, Kan^{r}, Sepc^{r}	2
Pn-*aqpC*P232Acom	ΔPn-*aqpC* strain with P232A mutated Pn-*aqpC* ectopically complemented, Kan^{r}, Sepc^{r}	2
Δ*hk07*	R6 strain with *hk07* deletion, Spec^{r}	This study
Δ*rr07*	R6 strain with *rr07* deletion, Spec^{r}	This study
ΔPn-*aqpC/hk07*	R6 strain with Pn-*aqpC* and *hk07* double deletion, Spec^{r}, Kan^{r}	This study
ΔPn-*aqpC/rr07*	R6 strain with Pn-*aqpC* and *rr07* double deletion, Spec^{r}, Kan^{r}	This study
WT-*strHgfp*	R6 strain with pPEPX1-P*strH-strHgfp*, Gent^{r}	This study
WT-*gh125gfp*	R6 strain with pPEPX1-P*gh125-gh125gfp*, Gent^{r}	This study
ΔPn-*aqpC-strHgfp*	ΔPn-*aqpC* strain with pPEPX1-P*strH-strHgfp*, Kan^{r}, Gent^{r}	This study
ΔPn-*aqpC-gh125gfp*	ΔPn-*aqpC* strain with pPEPX1-P*gh125-gh125gfp*, Kan^{r}, Gent^{r}	This study
Δ*rr07-strHgfp*	Δ*rr07* strain with pPEPX1-P*strH-strHgfp*, Spec^{r}, Gent^{r}	This study
ΔPn-*aqpC/rr07-strHgfp*	ΔPn-*aqpC/rr07* strain with pPEPX1-P*strH-strHgfp*, Kan^{r}, Spec^{r}, Gent^{r}	This study
ΔPn-*aqpC/rr07-strHgfp*com*rr07*	ΔPn-*aqpC/rr07-strHgfp* strain with *rr07* ectopically complemented on PIB166, Kan^{r}, Spec^{r}, Gent^{r}, Cm^{r}	This study
ΔPn-*aqpC*/*rr07-strHgfp*com*rr07*D55A	ΔPn-*aqpC/rr07-strHgfp* strain with *rr07*D55A ectopically complemented on PIB166, Kan^{r}, Spec^{r}, Gent^{r}, Cm^{r}	This study
WT-*rr07*-6His	R6 strain with 6 histidine-tagged *rr07*, Spec^{r}	This study
ΔPn-*aqpC-rr07*-6His	ΔPn-*aqpC* strain with 6 histidine-tagged *rr07*, Kan^{r}, Spec^{r}	This study
WT-strHgfp-rr07over	WT-*strHgfp* strain with *rr07* ectopically complemented on pIB166, Gent^{r}, Cm^{r}	
*rr07*over	R6 strain with *rr07* ectopically complemented on pIB166, Cm^{r}	This study

***E. coli* strains**		
DH5α	*supE44 lacU169* (80 *lacZ*M15) *hsdR17 recA1 endA1 gyrA96 thi-1 relA1 luxS*	TransGen Biotech, Beijing, China
BL21(DE3)	F- *ompT hsdS* (rBB- mB-) gal dcm (DE3)	TransGen Biotech, Beijing, China
**Plasmids**		
pIB166	*E. coli*-streptococci shuttle plasmid, Cm^{r}	3
pPEPX-Plac	Pneumococcus integrative plasmid, Spec^{r}	Addgene #122632
pPEPX1	pPEPX-Plac with Gent^{r}	This study
pET-28a	Protein expression plasmid, Kan^{r}	TransGen Biotech, Beijing, China
pIB166Pro-*rr07*	pIB166 carrying Pro-*rr07* DNA fragment, Cm^{r}	This study
pIB166Pro-*rr07*D55A	pIB166 carrying Pro-*rr07*D55A DNA fragment, Cm^{r}	This study
pPEPX1-*strHgfp*	pPEPX1 carrying P*strH*-*strHgfp* DNA fragment, Gent^{r}	This study
pPEPX1-*gh125gfp*	pPEPX1 carrying P*gh125*-*gh125gfp* DNA fragment, Gent^{r}	This study
pET-28a-*rr07*	pET-28a carrying *rr07* encoding gene, Kan^{r}	This study

[a], Kan^{r}, kanamycin; Spec, spectinomycin; Gent, gentamycin; Cm^{r}, chloramphenicol.

Table S2. Primers used in this study[a]

Primers	Sequence (5’-3’)	Purposes
*rr07*upF	CTCATGGATATCAGGTTCAGGTG	*rr07* deletion
*rr07*upNheIR	AATAT*GCTAGC*ATACATTTTCTCCCTTTCTAC	*rr07* deletion
*rr07*dnNheIF	AATAT*GCTAGC*TAAGATTTGTATTCCTTTAC	*rr07* deletion
*rr07*dnR	GGCAGTATTGGTTATTAAAGTTACGATTTCC	*rr07* deletion
*hk*07upF	CCTTGTATTTTCCTAAATGAGCTACTCC	*hk07* deletion strain
*hk*07upNheIR	AATAT*GCTAGC*TCTATTTTGTCGCCAATTTTTCATG	*hk07* deletion
*hk*07dnNheIF	AATAT*GCTAGC*CAAGATGAGTAGAAAGGGAG	*hk07* deletion
*hk*07dnR	GAGATGTTCAAATAAATGGAAAACATC	*hk07* deletion
aad9upNheIF	AAATT*GCTAGC*ATCAAAATAGTGAGGAGGATATATTTG	Spectinomycin resistance gene amplification
aad9upNheIR	AAGAC*GCTAGC*TTATAATTTTTTTAATCTG	Spectinomycin resistance gene amplification
OverproF	GAATTGTCTCCTGATAATTTTTTCAC	Promoter fragment amplification for Pro-rr07 construction
OverproR	CTAATAATACTTTATACATGATATTCCCCTAAAAATAAC	Promoter fragment amplification for Pro-*rr07* construction
Overrr07F	GTTATTTTTAGGGGAATATCATGTATAAAGTATTATTAG	*rr07* gene amplification for Pro-*rr07* construction
Overrr07R	TTAATGATGATGATGATGATGTAGTATAGTTTC	*rr07* gene amplification for Pro-*rr07* construction

PIBgibF	GAATTCTAGAGCTCGAGATCTATC	Gibson assembly of pIB166 and pro-*rr07*
PIBgibR	GTACCGCGGGGATCCAACAT	Gibson assembly of pIB166 and pro-*rr07*
Prorr07gibF	ATGTTGGATCCCCGCGGTACTAATAAATGCCTCATTTCACAATTAG	Gibson assembly of pIB166 and pro-*rr07*
Prorr07gibR	GATCTCGAGCTCTAGAATTCTTAATGATGATGATGATGATGATG	Gibson assembly of pIB166 and pro-*rr07*
Prorr07D55AF	CGATGTCATCATTTCCGCTGTCAATATGCCAGACA	For D55A mutation of *rr07*
Prorr07D55AR	TGTCTGGCATATTGACAGCGGAAATGATGACATCG	For D55A mutation of *rr07*
*strH*EcoRIF	ATAT*GAATTC*GAAGAAAGCCTGAGCCTAATC	Amplification of *strH* gene with its inherent promoter
*strH*BamHIR	ATAT*GGATCC*GTCTTCTTTTCTTTTAGTGAGTC	Amplification of *strH* gene with its inherent promoter
*gfp*BglIIF	ATAT*AGATCT*GGTGGTGGTGGTTCTGGTGGTGGTGGTTCTATGCGTAAAGGCGAAGAGCTG	*gfp* gene integrated into pPEPX1
*gfp*BamHIR	ATAT*GGATCC*TCATTTGTACAGTTCATCCATACCATGC	*gfp* gene integrated into pPEPX1
*gh125*EcoRIF	ATAT*GAATCC*AGTCTTTCCTTTAGCTGTTTTTC	Amplification of *gh125* gene with its inherent promoter
*gh125*BglIIR	ATTC*AGATCT*GCGGATATCCAAGTAATCC	Amplification of *gh125* gene with its inherent promoter
getaupF	GTTATTGCAATAAAATTAGCTTAGGTGGCGGTACTTGG	Gibson assembly of Gentamycin resistance gene into pPEPX-Plac
getaupR	ATAATCAACGAGGTGAAATCATGTTACGCAGCAGCAAC	Gibson assembly of Gentamycin resistance gene into pPEPX-Plac

F-pepx	GATTTCACCTCGTTGATTATG	Gibson assembly of Gentamycin resistance gene into pPEPX-Plac
R-pepx	GCTAATTTTATTGCAATAACAAG	Gibson assembly of Gentamycin resistance gene into pPEPX-Plac
rr07-6HisF	CAAGGTTCCTTCACCATTCCCTACTATG	*rr07*-6His strain construction
rr07-6HisBamHIR	CG*GGATCC*TTAATGATGATGATGATGATGTAGTATAGTTTCTACCTGTTTTCG	*rr07*-6His strain construction
*rr07*dnBamHIF	CGC*GGATCC*GATTTGTATTCCTTTACAAAAGGTGC	*rr07*-6His strain construction
*rr07*dnR	GTTACGATTTCCTGACCCAACCACACC	*rr07*-6His strain construction
*gh125*RTF	GATCTGGGAGCGCAAGTATGAG	For qRT-PCR
*gh125*RTR	AAGGAGAGTTCTTGTGGTCTTG	For qRT-PCR
*strH*RTF	CGATTGATCAAGCCATTGCTAAACTTCAAG	For qRT-PCR
*strH*RTR	GAATATCCGAGCTCACTGGCCT	For qRT-PCR
*gh92*RTF	GCAACAATGGTTTCTGGGATAC	For qRT-PCR
*gh92*RTR	TATCTGCGATAATGCCATCTAGC	For qRT-PCR
*gh29*RTF	TTCAATCGGAGAGGCAGATGTTTC	For qRT-PCR
*gh29*RTR	GGTCGCAAATTCATAAGGTCGTTC	For qRT-PCR

spr0081RTF	GGTTTAGTTGTATCTGTAGGGATTGC	For qRT-PCR
spr0081RTR	AATTGACTTCTTTGAGACCAAGAC	For qRT-PCR
*rr07*RTF	CCCCTAGATGGTTTAGTCGTTACAC	For qRT-PCR
*rr07*RTR	CAAGAACTACCTTCTCCAAGAGATTTAACT	For qRT-PCR
*pfl*RTF	CACCGTGTTACTAAGATGGACTACC	For qRT-PCR
*pfl*RTR	ATGAGATACAGCTCATTTCACCATATCC	For qRT-PCR
*ccpA*RTF	GTCGTGATTCCAAATATTACCAATGG	For qRT-PCR
*ccpA*RTR	CTGAGCGAATTTTATCTGTC AAGTG	For qRT-PCR
*rr07*pet28NcoIF	AATA*CCATGG*AAATGTATAAAGTATTATTAGTAGATG	RR07expression in pET28a
*rr07*pet28XhoIR	ATATA*CTCGAG*TAGTATAGTTTCTACCTGTT TTCGG	RR07expression in pET28a
*gh125*EMSA-F	CACTGGTCTGGGGCACAAAGT	For *gh125* EMSA
*gh125*EMSA-R	CGTGCAATTCCCTCTTTCAAACAAGTTT	For *gh125* EMSA
*strH*EMSA-F	GCTTATTATACCACACCAAAAAGGTAGC	For *strH* EMSA
*strH*EMSA-R	TGCAAAACCTCCTGATTGCATTGTTATATTG	For *strH* EMSA

[a], Italic nucleotide bases indicate restriction enzyme digestion sites.

References

1. Wang J, Wang W, Wang L, Zhang G, Fan K, Tan H, Yang K. 2011. A novel role of 'pseudo'γ-butyrolactone receptors in controlling γ-butyrolactone biosynthesis in *Streptomyces*. *Mol Microbiol* 82:236-250.

2. Hu Q, Tong H, Wang J, Ge P, Zhu L, Liu C, Zhang JR, Dong X. 2021. A novel aquaporin subfamily imports oxygen and contributes to pneumococcal virulence by controlling the production and release of virulence factors. *mBio* 12:e0130921.

3. Biswas I, Jha JK, Fromm N. 2008. Shuttle expression plasmids for genetic studies in *Streptococcus mutans*. *Microbiology (Reading)* 154:2275-2282.
